# The BioLexicon: a large-scale terminological resource for biomedical text mining

**DOI:** 10.1186/1471-2105-12-397

**Published:** 2011-10-12

**Authors:** Paul Thompson, John McNaught, Simonetta Montemagni, Nicoletta Calzolari, Riccardo del Gratta, Vivian Lee, Simone Marchi, Monica Monachini, Piotr Pezik, Valeria Quochi, CJ Rupp, Yutaka Sasaki, Giulia Venturi, Dietrich Rebholz-Schuhmann, Sophia Ananiadou

**Affiliations:** 1 School of Computer Science, University of Manchester, Oxford Road, Manchester, M13 9PL, UK; 2 National Centre for Text Mining, Manchester Interdisciplinary Biocentre, University of Manchester, 131 Princess Street, M1 7DN, Manchester, UK; 3 Manchester Interdisciplinary Biocentre, University of Manchester, 131 Princess Street, M1 7DN, Manchester, UK; 4 Istituto di Linguistica Computazionale del CNR, Via Moruzzi 1, 56124 Pisa, Italy; 5 European Bioinformatics Institute, Wellcome Trust Genome Campus, Hinxton, Cambridge, CB10 1SD, UK; 6 Toyota Technological Institute, Nagoya, Japan

## Abstract

**Background:**

Due to the rapidly expanding body of biomedical literature, biologists require increasingly sophisticated and efficient systems to help them to search for relevant information. Such systems should account for the multiple written variants used to represent biomedical concepts, and allow the user to search for specific pieces of knowledge (or *events*) involving these concepts, e.g., protein-protein interactions. Such functionality requires access to detailed information about words used in the biomedical literature. Existing databases and ontologies often have a specific focus and are oriented towards human use. Consequently, biological knowledge is dispersed amongst many resources, which often do not attempt to account for the large and frequently changing set of variants that appear in the literature. Additionally, such resources typically do not provide information about how terms relate to each other in texts to describe events.

**Results:**

This article provides an overview of the design, construction and evaluation of a large-scale lexical and conceptual resource for the biomedical domain, the BioLexicon. The resource can be exploited by text mining tools at several levels, e.g., part-of-speech tagging, recognition of biomedical entities, and the extraction of events in which they are involved. As such, the BioLexicon must account for real usage of words in biomedical texts. In particular, the BioLexicon gathers together different types of terms from several existing data resources into a single, unified repository, and augments them with new term variants automatically extracted from biomedical literature. Extraction of events is facilitated through the inclusion of biologically pertinent verbs (around which events are typically organized) together with information about typical patterns of grammatical and semantic behaviour, which are acquired from domain-specific texts. In order to foster interoperability, the BioLexicon is modelled using the Lexical Markup Framework, an ISO standard.

**Conclusions:**

The BioLexicon contains over 2.2 M lexical entries and over 1.8 M terminological variants, as well as over 3.3 M semantic relations, including over 2 M synonymy relations. Its exploitation can benefit both application developers and users. We demonstrate some such benefits by describing integration of the resource into a number of different tools, and evaluating improvements in performance that this can bring.

## Background

Automatic literature analysis and text mining has developed into a discipline of bioinformatics research. As the need for biomedical text mining systems grows [1-3], the requirement for domain-specific lexical resources [4] that can aid systems in accurately identifying and extracting knowledge embedded within texts is becoming stronger.

The identification of knowledge includes, but is not limited to, the recognition of concepts. Rather, knowledge consists of relationships that hold between these concepts. Such relationships (or *events*) may describe, for example, an interaction between 2 proteins, or the regulation of a gene by a protein, etc. Within texts, events are frequently organized around verbs. A text mining system that allows users to search for events as well as individual concepts provides the means to locate information much more quickly and efficiently than traditional keyword-based methods. Advocates of systems that take verbs into account include, notably, Bill Gates, who has stated that *The future of search is verbs *(as reported by E. Dyson at Big Think's *Farsight 2011: Beyond the Searchbox *event [5]).

Since knowledge is expressed by words, text mining systems can benefit from access to resources that provide precise information about how words behave in texts within their domain of operation. Within the biomedical field, the recognition of both concepts and events present their own challenges, which must be considered during the construction of such a resource.

Concepts are represented in text by biomedical terms. Inventories of such terms can already be found in a number of well-established reference data resources, which are subject to ongoing improvements. These include the Gene Ontology [6], ChEBI [7,8], BioThesaurus [9] and the NCBI taxonomy [10]. However, in the context of text mining systems, the use of such resources as an aid to recognizing terms in biomedical texts presents a number of drawbacks:

• Each resource generally has a different focus. For example, BioThesaurus covers gene and protein names, while ChEBI deals with chemical entities of biological interest. As the resources are not linked together [11], the coverage of biological terminology contained within them remains dispersed, making it difficult to use them in wide-coverage or re-targetable text mining systems.

• The resources are often completely manually curated and oriented towards human use [12,13], meaning that labels for concepts contained within them deviate from the language used by the scientist in the scientific literature. This is a barrier for efficient text mining solutions, which need to know precisely which concepts manifest themselves in texts, and how. In biomedical texts, terms frequently have a number of different written forms (called "variants") [14]. The set of possible variants of a given term is constantly changing, since new variants are frequently coined by authors. This makes it difficult for resources that are completely manually curated to keep track of all variants that may appear in the literature. Consequently, determining exactly which words in a text correspond to biomedical terms, and moreover, which concepts they represent (in terms of, e.g., database identifiers such as a UniProt [15] accession number), is a major challenge, which cannot be fully supported by existing resources.

In terms of recognising events expressed in biomedical texts, the challenge presents itself in terms of the idiosyncratic patterns of behaviour of individual verbs, which occur in terms of both grammar (i.e., which terms/phrases in a sentence are linked to the verb, and how) and semantics (i.e., the meaning/role of each linked term/phase in relation to the knowledge being expressed). The ability of a system to extract events accurately requires access to both types of information. Until now, only a small number of attempts have been made to create resources that describe the behaviour of verbs in biomedical texts, all of which have shortcomings. These shortcomings include being too small scale to be of practical use (e.g., [16,17]), covering only grammatical and not semantic behaviour, and not taking sufficient account of real, observed usage of the verbs within texts (e.g., [18]).

We have responded to the issues outlined above by constructing the BioLexicon, which is a standards-compliant, reusable, lexical and conceptual resource that has been designed to support a range of tasks performed by biomedical information retrieval and text mining systems. The main criteria in constructing the lexicon were as follows:

• It should describe real, observed usage of words in biomedical texts, in order to facilitate sophisticated text mining applications.

• It should be a large-scale and wide-coverage resource, to facilitate its use in a broad spectrum of tasks applied to biomedical texts.

• It should include a wide range of variants of biomedical terms appearing in biomedical literature.

• It should include linguistic information, including both the grammatical and semantic behaviour of a range of domain-relevant verbs.

The BioLexicon is intended to be integrated into systems, in order to facilitate improved or more advanced behaviour. As such, whilst the direct users of the BioLexicon will be system developers, the end-users of implemented systems can be seen as indirect users, in that they will benefit from the enhanced system functionalities that are provided through the use of the BioLexicon.

The main purpose of this article is to provide a general overview of the BioLexicon, covering four main areas:

• The design considerations and challenges faced in building the BioLexicon.

• The methodology employed to build the lexicon in response to these considerations and challenges.

• Provision of information about the coverage of the lexicon, and the types of information contained in typical entries.

• A description of how the BioLexicon has been exploited through its integration into a number of tools relating to biomedical information retrieval and text mining.

### BioLexicon overview

The BioLexicon contains ~2.2 M lexical entries for biomedical text mining, with information regarding 4 part-of-speech (POS) categories, i.e. nouns, verbs, adjectives and adverbs; each category includes both domain-specific terms and general language words. The construction of the lexicon has involved the employment of a range of methods, both manual and automatic. Automatic methods have been employed in cases where the very large scale of the task would make a manual solution impractical or extremely costly, and where the performance of the automated solution is of a sufficiently high quality.

The construction criteria outlined in the previous section have been achieved in a number of ways, including the following:

• **Real, observed usage of words **- A large amount of information about domain-specific words has been derived directly from corpora of biomedical texts, to ensure that actual usage is reflected in the entries of the BioLexicon. General language vocabulary is accounted for in the BioLexicon through the inclusion of entries from the MedPost dictionary [19]. Since the vocabulary in this dictionary is drawn directly from MEDLINE abstracts, it also reflects real vocabulary usage within biomedical texts.

• **Large-scale and wide-ranging coverage **- The MedPost dictionary contains some 10,000 entries, which constitute almost 93% of the words appearing in MEDLINE abstracts. In the BioLexicon, the entries from the MedPost dictionary are supplemented with terms extracted from 14 different biomedical resources. Since each resource has a different focus, as detailed in the *Methods *section, the BioLexicon constitutes a unified, consolidated resource of biomedical terminology.

• **Term variants **- Terms extracted from existing resources are augmented with gene and protein name variants, automatically extracted from 15 million MEDLINE records, using text mining methods.

• **Linguistic information **- The Biolexicon includes comprehensive information about the behaviour of verbs. This includes

○ Grammatical information, which has been automatically acquired from a biomedical text corpus of 6 million words.

○ Semantic information, which is based on manual annotation of events by biology experts in 677 MEDLINE abstracts.

○ Manually-determined links between grammatical and semantic information.

Care has been taken to ensure that the quality of the entries in the BioLexicon is as high as possible. Steps taken in this respect include the following:

• Employment of measures to verify the quality of entries extracted from existing biomedical resources.

• Use of state-of-the-art text mining tools to perform automatic processing steps

• Post-filtering of automatically extracted information.

• Verification of the quality of the manually-produced annotations from which verbal semantic information is derived, through double annotation of a proportion of the abstracts by different annotators, and calculation of inter-annotator consistency rates.

The BioLexicon has already been integrated into a number of different domain-specific tools, i.e. a part-of-speech tagger, a lemmatizer, an information extraction system and a fact extraction system. The use of the BioLexicon within these tools, together with some evaluation data, is provided in the *Discussion *section. Further examples of tasks that could be supported by the BioLexicon include the following:

• Dynamic query term completion during search input. When the user has started to type into the search box, suggestions of possible completions of query terms can be made automatically by the system, through reference to BioLexicon entries.

• Detection of protein-protein interactions (PPI) via co-occurrence. Some PPI detection methods are based on calculating co-occurrence statistics between pairs of proteins within, e.g., sentences or abstracts, e.g. [20]. The BioLexicon can provide support in the recognition of proteins to facilitate this task.

• Identification of associations between different types of terms, in a similar way to [21]. Given a search term, associations can be found by calculating which other types of terms (e.g. diseases, drugs, etc.) frequently co-occur with the search term (e.g., in the same abstract). Finding such associations can be useful in allowing researchers to answer questions such as *Which diseases are relevant to a particular gene? *The BioLexicon can provide support in the recognition of relevant co-occurring terms, as well as providing variants of the entered search term, in order to expand the results returned by the query.

The complete BioLexicon is available in a relational database format from the European Language Resources Association (catalogue reference T0373: http://catalog.elra.info/product_info.php?products_id=1113). A part of the data in the BioLexicon, i.e., the repository of terms that have been extracted from existing databases (thus excluding the many other variants found in text, that are however incorporated in the full BioLexicon), is freely available for download in XML [22] format from http://www.ebi.ac.uk/Rebholz-srv/BioLexicon/biolexicon.html. The content of the BioLexicon can also be queried using a web interface available at: http://wiki.ilc.cnr.it/BootStrep/searchPanel.action.

The BioLexicon was developed as part of the BOOTStrep project, which had the aim of developing resources and text mining tools that could boost the performance of various biomedical application tasks. Whilst the coverage of the BioLexicon (particularly biomedical terms) is intended to be wide enough to allow it to be used as a general-purpose resource for the biomedical literature, gene regulation was chosen as the domain that would receive a particular focus during the project. Therefore, special attention has been paid to ensuring that this domain is covered comprehensively in the BioLexicon. In line with the focus on gene regulation, another major outcome of the project was the Gene Regulation Ontology (GRO) [23]. The GRO is a conceptual model for the domain of gene regulation, which covers processes that are linked to the regulation of gene expression, as well as physical entities that are involved in these processes (such as genes and transcription factors). It incorporates other well-established biomedical ontologies, such as the Gene Ontology [6] and the Sequence Ontology [24]. It is listed on the Open Biomedical Ontologies [25], demonstrating its commitment to adopting developmental best practices, in order to foster interoperability. Whilst the GRO and BioLexicon can function independently of each other, a mechanism has been provided to link lexical entries in the BioLexicon to ontological classes in the GRO. The combined capacity of these resources can facilitate advanced information extraction and text mining capabilities.

This article focusses primarily on the two areas of the lexicon that support the key tasks of automatic biomedical term extraction and knowledge extraction from text, namely domain-specific nouns and verbs. We cover both the acquisition of biomedical terms and their variants, and the acquisition of grammatical and semantic behaviour of verbs. Particular attention is paid to verbs for a number of reasons. Firstly, as described above, they play a crucial role in the development of advanced search systems that allow pieces of knowledge (i.e., events) to be extracted and searched. Secondly, given the idiosyncratic nature of verb behaviour, and the fact that both grammatical and semantic information are required for the accurate extraction of events, the process of acquiring information for verbs is more complex than for terms. Finally, we have applied novel methods to acquire much of the verbal information.

The remaining part of this section describes the main design considerations that were taken into account during the construction of the BioLexicon. Focussing on the application areas of automatic biomedical term extraction and knowledge extraction, the following points are addressed:

• Existing problems and challenges faced in each application area.

• A description of how these application areas can be improved by the availability of enhanced resources.

• Difficulties faced in creating such enhanced resources, together with proposed solutions.

### Biomedical term extraction

The use of biomedical terms as references to concepts and entities in biomedical data resources is paramount for the integration of the literature into the bioinformatics data infrastructure. Knowledge of as many term variants as possible can be a huge asset to text mining systems, since searching of biomedical texts can be vastly improved if all variants of some term are automatically resolved to the same entity. Importantly, this means that the biologist does not need to be concerned with the often impossible task of trying to enumerate all variants of a particular term as part of their search. Rather, he only has to enter a single variant of each search term, in response to which the enhanced information retrieval system is able to return not only documents that mention the entered search term, but also those documents containing its known variants. Similarly, a text analysis solution would suggest only one canonical entity to the biologist for all alternative variants found in the text.

The problems of discovery, mapping and integration of new terms and their variants into existing biomedical resources have constituted a major challenge for biomedical text mining systems [26,27]. This task is particularly challenging, due to the potentially large number of variants, and the range of different forms that these variants can take. Some common types of variant forms are as follows:

• Orthographical, e.g.,

○ spelling variations (*tumour *vs. *tumor*)

○ hyphens/slashes (*amino acid *vs. *amino-acid*)

○ case variations (*NF-KB *vs *NF-kb*)

• Morphological (e.g. *Parkinson disease *vs *Parkinson's disease)*

• Synonyms (e.g. *carcinoma *vs. *cancer*)

• Acronyms (e.g. *Interleukin-2 *vs. *IL-2*)

• Structural (e.g. *NF-kappa-B inhibitor *vs. *Inhibitor of NF-kappa-B*)

• Semantic, i.e., variants whose textual form is unrelated, but which nevertheless denote the same entity (*Ksp1 protein *vs. *Ppk20*)

#### Domain-specific nouns

In the BioLexicon, biomedical terms constitute the major part of the domain-specific nouns. As mentioned above, these terms have been gathered together from several existing terminological databases and ontologies, in an attempt to construct a large-scale and unified repository of biomedical terms, which is suitable for use by wide-coverage text mining systems. In order to reflect more closely the actual occurrence of these terms in biomedical texts, i.e., to account for the many different variants that can appear, Named Entity Recognition (NER) techniques have been applied to extract additional variants from 15 million MEDLINE records. This automatic procedure can be re-run at regular intervals to ensure that the BioLexicon contains the most up-to-date terms variants.

NER is a process that recognises Named Entities (NEs) of interest in text [3], such as genes or proteins in the biomedical domain. It does this by considering features of the entities, such as:

• The POS of their constituent words, e.g., noun, adjective, etc.

• Whether the words start with a capital letter.

• Whether part or all of the candidate term is already a known term (which requires access to a repository of biomedical terms).

• Which words occur in the vicinity of the term, etc.

NER is normally carried out either through the application of a set of rules or by applying machine learning techniques to a set of documents in which NEs have already been manually annotated, in order to determine the set of features that can most accurately predict the desired entities of interest in a text.

### Biomedical knowledge extraction

As an example of a biomedical event organized around a verb, consider the following simple sentence:

*Fis activates rrnB P1* - Existing terminological resources may contain sufficient information to identify *Fis *and *rrnB P1 *as relevant terms, and furthermore may categorise their semantic types as *PROTEIN *and *PROMOTER*, respectively. However, such resources generally lack the detailed information about verbs that would be required to extract the relationship between these two terms and their involvement in the same event, namely the positive regulation of a gene [23,28,29]. Text mining systems should be able to extract this knowledge in a structured format, such as the following:

• There is an event of type POSITIVE_REGULATION, organized around the verb *activates*.

• The event has 2 participants, namely:

○ *Fis*, which is the causer of the event (we call this the AGENT of the event).

○ *rrnB P1*, which is affected by the event (i.e., the THEME of the event).

Such structured knowledge facilitates advanced, event-based searching [30,31], which is much more powerful and specific than simple keyword searching.

Suppose that a biologist wishes to find which promoters are positively regulated by the protein *Fis*. Using a simple keyword search, the biologist could enter the search terms *Fis *and *activate *(a verb amongst others that is frequently used to represent events of positive regulation in molecular biology). Carrying out the search in this way presents a number of problems, for example:

• Relevant documents should contain a sentence in which *Fis *is grammatically related to the verb *activate *(i.e., *Fis *should be the subject). However, a simple keyword search does not allow such specific criteria to be specified. The search may even return documents in which the two terms occur in separate sentences. Thus, many irrelevant documents are likely to appear in the search results.

• Positive regulation events may be organised around verbs other than *activate*, e.g. *stimulate*. As it is difficult to determine the exact range of verbs that can describe such events, formulating a query that will return all relevant events is problematic.

In contrast to keyword searching, event-based searching carries out searches over structured *events *that have been extracted from text. This means that it is not necessary to be concerned about the exact way in which the event is specified in the text (including the choice of verbs and the order of the terms in relation to the verb). As events and their participants are grounded to actual words in the text, users can still review search results in the familiar way, i.e., by viewing a snippet of text from each document containing the relevant event [32].

In order to perform event-based search, users can partially complete a template that specifies constraints regarding the events to be retrieved. For the search problem introduced above, the biologist may specify the following semantic constraints, to ensure that the only documents returned by the search are those containing events describing promoters that are positively regulated by the protein *Fis*:

• The event type should be POSITIVE_REGULATION.

• The event should have an AGENT, which has the value *Fis *and has the type PROTEIN

• The event should have a THEME, with the type PROMOTER.

Semantic constraints can be specified in more or less general terms depending on the requirements of the search, e.g., it is possible to use specific keywords as the values of event participants, but it is equally possible to specify more general types, e.g., *PROMOTER*, which could correspond to a wide range of possible values.

The semantic event structure above introduces a generalisation of events and allows for deviation from the way the events are represented in text. However, in order to extract instances of events from texts, text mining systems need to determine how to map from the textual representation of the event to the more abstract, structured format on which queries can be performed. This requires detailed information about the patterns that can occur in text to describe relationships between terms.

Verbs are central to many of these event representation patterns and, in most cases, the semantic event participants will be grammatically related to the verb. The automatic recognition of events thus requires the following to be determined:

• Which verbs describe relevant events.

• For each relevant verb:

○ Grammatical structure - which terms and other phrases constitute participants of the event. This is determined by locating those phrases that have a grammatical relationship to the verb (e.g., subject or object). Such phrases are called the *arguments *of the verb.

○ Semantic structure - how each identified event participant contributes towards the description of the event (using *semantic roles *such as AGENT and THEME).

As mentioned above, individual verbs have idiosyncratic properties, with differing grammatical and semantic structures. The number of typical grammatical arguments (e.g. subject and object, as well as other possible argument types) varies from verb to verb, as does the typical semantic interpretation of a given argument. Such properties of individual verbs, which are described in further detail below in the *Domain-specific verbs *subsection, motivate the need for a large-scale domain-specific vocabulary resource that includes explicit linguistic information that can help text mining systems to determine how terms and concepts are used within texts [33].

#### Domain-specific verbs

In the BioLexicon, domain-specific verbs have been manually selected, based on an examination of biomedical literature. Since patterns of behaviour are often verb-specific, it is not possible to predict semantic patterns based on grammatical patterns. For these reasons, a lexical resource conceived to support biomedical text mining applications effectively must include separate descriptions of both the grammatical and semantic behaviour for individual verbs, as well as providing the links between the two types of patterns. The information about patterns is represented in a template-like structure called a *frame*.

Each verb may have more than one frame, corresponding to different possible patterns of grammatical and/or semantic behaviour. As frames are abstract specifications that give predictions [34], it may be that, in a particular sentence, a predicted argument or participant is not present. There is thus optionality built into frames. This is useful in helping systems to deal with common styles of writing in scientific text, in which passive constructions are often preferred to active ones [35], e.g., *x was activated*, where the writer does not overtly state the AGENT, i.e., who or what was responsible for the action. It is furthermore useful in helping a system to infer what kind of AGENT would typically be responsible, when that AGENT is missing. By applying the predictions (or constraints) specified in frames, a text mining system can deliver more accurate and complete analyses, by matching a frame specification to instances in the text [36].

#### Grammatical argument patterns for verbs

In terms of possible grammatical argument patterns, transitive verbs such as *activate *or *regulate *take direct objects (*The phosphorylated form of enzyme IIAGlc probably activates **adenylate cyclase***), whilst intransitive verbs such as *act *or *compete *do not. Some verbs (e.g. *show*, *demonstrate*) permit a that-complement clause (as in *The analysis shows **that **..*.), whilst others take an infinitival clause (e.g. *need*, *act *as in *Bisulfite acts **to inhibit excision repair***).

The availability of grammatical information is particularly important in extracting events from sentences containing multiple verbs, when it must be determined which words and phrases relate to which verbs. As an example, consider the following sentence, in which there are 3 verbs describing events (emboldened), each of which has a different set of participants: *IHF may **inhibit **ompF transcription by **altering **how OmpR **interacts **with the ompF promoter*.

#### Semantic argument patterns for verbs

On the semantic side, variations can occur both in the patterns of the semantic roles that apply to event participants and the types of entities (i.e., NE types) that constitute the participants. Many verbs include among their arguments both an AGENT and a THEME. This is exemplified in the sentence *The enzyme can bind**** a second DNA duplex***, in which *the enzyme *and *a second DNA duplex *play the semantic roles of the AGENT and THEME of the binding event, respectively.

In contrast, some verbs do not specify an AGENT at all, as in the case of the verb *accumulate *in the sentence, ***Neither free rRNA nor free r-protein **accumulate in appreciable amounts during balanced growth*, in which the emboldened phrase is affected by the event expressed by the verb, and thus plays the role of the THEME.

The above examples demonstrate the lack of consistent correspondence between grammatical and semantic arguments. Depending on the verb, the grammatical subject may represent, in semantic terms, the AGENT or the THEME of the event expressed by it. In contrast, different grammatical forms can be used to express the same semantic role. For example, the THEME of an event can be associated with either the grammatical subject or object.

Other types of semantic roles are also possible, as in the sentence *Bisulfite acts **to inhibit excision repair***. Whilst *Bisulfite *is clearly the AGENT, it would be incorrect to say that the underlined argument is the THEME of the event. Rather, according to our inventory of semantic roles (described in the *Methods *section) it should be labelled as the PURPOSE of the event.

According to the verb in question, a particular event participant (i.e., having a specific semantic role) can correspond to different NE types. For example, the AGENT of an event organised around the verb *bind *is generally an entity with the NE type PROTEIN, whilst for events that are organised around the verb *encode*, the AGENT is normally an entity with the NE type DNA. The availability of such information can be useful as a filter when extracting events, ensuring that only events whose participants correspond to the expected NE categories are stored as relevant events. This is particularly useful in the case of general language verbs, which, according to the types of participants involved, may or may not describe biologically relevant event.

#### Resources providing grammatical and semantic information about verbs

For general (i.e., non-specialist) English language, resources called computational lexicons have been created (e.g., [37-39]) that contain the above types of information for verbs, thus allowing relationships between terms and phrases within sentences to be identified automatically. To some extent, it is possible to employ these existing resources in text mining systems that operate in the biomedical domain [40,41]. However, it is strongly beneficial for text mining systems to have access to domain-specific counterparts of these general language resources, for a number of reasons. Firstly, there are verbs in biomedical texts that are unlikely to appear in general language computational lexicons, e.g., *methylate*, *phosphorylate*, etc., either because they are specific to the domain or they are used infrequently in the general language domain. Secondly, in contrast, commonly-occurring verbs whose behaviour is described in general language resources may have different grammatical or semantic properties within the biomedical domain. For example, the verb *activate*, when used in the context of activating a bank account, has a very different meaning and different grammatical patterns to when it is used in the context of biomedical literature.

In biomedical texts, it is common for verbs to take an extended number of arguments, as compared to general language texts, often in the form of adverbs, e.g., *rapidly*, or phrases beginning with prepositions, e.g., *in E. coli*. In general language, such phrases are not typically tightly connected with the verb. However, in the biomedical domain, there are often stronger connections to the verb, in that these phrases correspond semantically to locations, manners, timings, rates and experimental conditions of biomedical events [42]. For example, when dealing with a mutation elevating expression in comparison to some wild-type, the rate of that elevation is highly important to the correct interpretation of the event. Thus, constructions such as the following are found: *X elevated expression of Y by 10-20 fold over Z*.

Using information about verbs from resources built for processing general language texts may thus result in the text mining system making incorrect analyses or interpretations, or indeed failing to achieve an analysis. Although some domain-specific extensions to the above resources have been attempted, i.e., PASBio [16,43] and BioFrameNet [17], they are currently very small scale (e.g., PASBio contains information for only 30 verbs).

To our knowledge, the only existing large-scale computational lexicon specifically developed for the biomedical domain is the SPECIALIST lexicon [18] (http://lexsrv3.nlm.nih.gov/LexSysGroup/Projects/lexicon/current/web/index.html), which contains both general English words and biomedical vocabulary, with a particular emphasis on medical and health-related vocabulary, extracted from resources such as the UMLS Metathesaurus [44] and Dorland's Illustrated Medical Dictionary. The SPECIALIST lexicon includes grammatical patterns for verbs, although it is not based on real, observed usage in texts, and the number of different grammatical patterns that are described is relatively small. In addition, no semantic information for verbs is included.

The lack of an existing resource that is suitably tailored to facilitating accurate extraction of events within the biomedical domain motivated our decision to undertake our own acquisition of grammatical and semantic frame information for verbs relevant to the domain. This procedure was carried out using a combination of manual and automatic techniques, and using domain-specific texts to ensure that the behaviour of the verbs stored in the lexicon reflects the way that they are actually used within the biomedical literature. These techniques are more fully described in the *Methods *section below.

The remainder of this paper is organised as follows. Firstly, in the *Methods *section, we describe in detail the methods by which the different types of entries in the BioLexicon have been collected and curated, with a particular focus on the most important features of the lexicon, namely the collection of the noun entries corresponding to biomedical terms, and the acquisition of grammatical and semantic information relating to domain-specific verbs. We also briefly describe the representation model of the lexicon. In the *Results and Discussion *section, we present statistics regarding the entries in the BioLexicon, compare its coverage to some existing resources, provide some sample entries in the context of the web interface, and demonstrate the utility of the lexicon by examining how it has been used to improve the performance of different text mining processes. Finally, in the *Conclusions *section, we summarise the main benefits of the BioLexicon to the biomedical text mining community and introduce some directions for future work.

## Methods

This section describes the main methods that have been used to create the entries in the BioLexicon. We begin with a description of how terms and concepts from disparate biological databases, controlled vocabularies and ontologies have been combined into a single, unified resource. We then continue by explaining how the many variants of these terms and concepts have been accounted for by applying text mining techniques to discover new variants of gene and protein names appearing in the literature. We next turn our attention to the entries in the lexicon corresponding to terminological verbs. These entries contain both the grammatical and semantic information that is required by systems aiming to determine the relationships that hold between concepts mentioned in texts. We explain the semi-automatic methods by which these types of information have been acquired from domain-specific collections of texts. Finally, we motivate and describe the model chosen to represent the BioLexicon, together with some details regarding its implementation.

### Gathering terms used in biomedical texts

The largest category of entries in the BioLexicon is nouns, which correspond mainly to biomedical terms, e.g., gene/protein names. These are gathered primarily from existing databases. However, text mining techniques have also been applied to abstracts extracted from approximately 15 million MEDLINE records (approximately 60% of the records have abstracts) to recognise new term variants and to map them to existing entries in the lexicon automatically.

#### Extracting terms from existing databases

Existing biological databases are characterized by a high coverage of biological entities, and contain terms annotated with widely recognized and interoperable accession numbers (e.g., UniProt). However, as terms in these resources are not necessarily intended to reflect the exact wording found in the scientific literature (for example, they may be formal concept or classification labels), some initial filtering of potential terms was necessary in the construction of the BioLexicon. As an example, terms referring to proteins identified in the course of high-throughput experiments such as *hypothetical protein *were ignored due to their low information value. Other indications of the discriminatory power of a term available in the BioLexicon include its frequencies of occurrence in MEDLINE [45] and in the British National Corpus [46]. These features have proven useful in identifying potentially polysemous terms (e.g., cases where a gene name may refer to a number of orthologous or otherwise homologous genes) during the NER task [47].

Terms in the BioLexicon are organised according to semantic category. The choice of semantic categories can be explained in two ways. Firstly, since one of the goals of the BioLexicon was to achieve maximum, general-purpose coverage of entities appearing in biomedical texts, common semantic types relevant to the biology domain were selected, i.e., gene and protein names, chemicals of biological interest and species names. Secondly, the inclusion of smaller, more focused sets for terms such as operon names or sequence ontology terms was intended to make the lexicon suitable for text mining applications dealing with the chosen specialist topic of gene regulation. For each semantic category, the corresponding terms have been extracted from specific resources, which may be either specialised databases, or particular categories within more general databases. Table 1 lists the different semantic categories for terms in the BioLexicon, together with the exact sources from which the terms within the category have been drawn. The complete list of sources used is as follows: Gene Ontology [6], Cell Ontology [48], OMIM (Online Mendelian Inheritance in Man) [49], ChEBI [7,8], Enzyme Nomenclature [50], Sequence Ontology [24], RegulonDB [51], CORUM [52], Operon Database (ODB) [53], BioThesaurus [9], the NCBI taxonomy [10], InterPro [54], TRANSFAC [55] and the IMR - INOH Protein name/family name Ontology [56].

**Table 1:** Sources of different types of terms in the BioLexicon

**Semantic type**	**Resources**	**Semantic type**	**Resources**
	
Cell	Cell ontology	Nucleic Acid Region	Sequence Ontology (Region)
	
Cell Component	Gene Ontology (GO:0005575 cellular component)	Operon	RegulonDB, ODB
	
Chemical	CHEBI (IMR:0000947 chemical)	Organism	NCBI Species
	
Disease	OMIM	Transcription Factor-Binding-Site	Sequence Ontology
	
Enzyme	Enzyme Nomenclature	Protein	BioThesaurus
	
Gene	BioThesaurus	Protein Complex	Corum database
	
Ligand	IMR - INOH Protein name/family name ontology	Protein Domain	InterPro
	
Nuclear Receptor	Gene ontology (GO:0004879 ligand-dependent nuclear receptor activity)	Transcription Regulator	RegulonDB, TransFac, Gene Ontology Annotation

Each semantic type included in the BioLexicon is shown, together with resource(s) from which it has been extracted. In the case of large resources containing several types of terms (e.g., the Gene Ontology), the exact category of the extracted terms within the source resource is shown, together with an identifier for that category, if available.

By far the largest categories of terms are gene and protein names, which have been gathered from BioThesaurus [9]. BioThesaurus consists of several million records of gene and protein names, extracted from several online resources. Although BioThesaurus is extremely comprehensive, and contains useful features, such as organizing variants of terms into sets of synonyms, it also contains a certain amount of noise. For example, in building the BioLexicon, a total of 2,972,035 terms were identified as nonsense names, which were too general to be of any practical value. In order to ensure the quality of entries extracted for inclusion in the BioLexicon, only the entries from BioThesaurus that could be mapped to SwissProt entries through a common UniProt accession number were retained. This is because the SwissProt section of the UniProt knowledgebase contains manually-curated protein sequence records.

#### Textual variants of terms

As explained above, the large number of variant terms that appear in texts can be a major barrier to text processing. Text mining systems should have the ability to locate all documents or events that mention a particular entity, regardless of which variants of that term are used in different documents.

In the existing resources from which terms have been gathered, known variants are assigned to a unique identifier (e.g., UniProt accession number). For example, the following 6 terms extracted from the BioThesaurus all have the same identifier: (Q8K4R9): *Hepatoma up-regulated protein, Hurp, hepatoma up-regulated protein, discs large homolog 7, "discs, large homolog 7" *and *Dlg7*. The BioLexicon retains such clusters of terms and builds upon them by finding new variants that appear in the literature (see next section). These enhanced clusters allow text mining systems making use of the resource to resolve any of these variants appearing in text to the same entity, and thus help to improve search results, no matter which variant is entered by the user.

#### Extracting biomedical term variants from text

In the BioLexicon, clusters of gene/protein names extracted from existing resources are augmented with further new variants that appear in biomedical texts. This is an important activity, to ensure that that the inventory of terms contained within the BioLexicon can deal with the wide variation of terms that is observed in biomedical texts. Such variation is particularly prevalent for gene and protein names, hence our decision to concentrate on these. Given the large and rapidly growing number of biomedical articles, the use of automatic methods is the most efficient and cost-effective way to discover a wide range of new term variants. Accordingly, a combination of different text mining techniques has been used to augment the BioLexicon with new term variants. NER is firstly used to locate candidate term variants in biomedical abstracts. These automatically extracted terms are assigned probabilities to allow them to be distinguished from original terms. NER is followed by a term mapping process, which finds appropriate term cluster(s) to which a newly discovered term can be mapped. In order to account for the regular appearance of new terms variants appearing in the literature, this automatic procedure can be run at regular intervals on newly published texts.

Prior to performing the term mapping process, gene/protein names are firstly automatically sub-clustered, based on soft string matching techniques, whereby similarity scores are calculated between terms based on commonalities between their textual forms [57]. This process is used to recognise several of the types of term variation listed the *Background *section, e.g., orthographical or morphological variations. The same soft string matching techniques are then applied to perform the term mapping process. This process involves finding the most appropriate term sub-cluster(s) to which each newly-discovered term can be mapped, i.e. the sub-cluster(s) whose members have the highest string similarity scores to the new term. This allows the newly discovered term to be assigned to the most appropriate UniProt accession number(s). As the same gene or protein name may be used for a number of orthologous or otherwise homogenous genes, a method based on static dictionary features [47] was developed to allow such potentially polysemous gene and protein names to be identified.

We have used an NER tool that employs a hybrid method [58], combining the use of a dictionary containing known gene/protein names with a statistical machine learning method. The dictionary consists of 266,000 entries for general English words extracted from WordNet [59], together with 1.3 million entries for protein names, extracted from BioThesaurus [9]. The tool is available at http://text0.mib.man.ac.uk/~sasaki/bootstrep/nemine.html.

Text mining techniques are typically evaluated against 'gold standards' [60]. We evaluated the NER tool against the JNLPBA-2004 dataset [61]. This was used in a shared task in a community evaluation challenge to compare the performance of NER systems operating in the biomedical domain. The evaluation of our NER tool against this dataset resulted in an F-score of 73.78. The F-score obtained was the second highest obtained for protein name recognition reported using the JNLPBA-2004 data set. Comparison with other recent experiments carried out on the same dataset, e.g. [62], demonstrate that this level of performance represents the state-of-the-art in protein name recognition. As has already been acknowledged by challenges such as BioCreAtIvE [63], automatic recognition of gene and protein names is a challenging task, due to factors such as extensive ambiguity, overlap of gene names with general English terms and complex multi-word terms. This means that achieving a high performance in NER for these entity types is a difficult task. One of the major aims of the BioLexicon was to construct a comprehensive resource that would help to remedy this situation.

We applied our NER system to abstracts extracted from approximately 15 million MEDLINE records (2006 release). Gene/protein names identified with a probability greater than 0.99 were then selected as candidates for new gene/protein variants. These candidates were only be added to the resource if there was sufficient similarity with an existing term, as determined by the term mapping process described below.

For efficiency reasons, the term mapping was conducted through term normalization. Since the lexicon contains about 2 million gene/protein names, straightforward similarity calculation of term pairs is not practical: when an NER component extracts tens of millions of gene/protein name candidates from the whole of MEDLINE, the similarity distance of 2 × 10^13 ^pairs of terms must be calculated. This amount of computation can however be drastically reduced to 10^7 ^normalizations and index lookups. The normalization steps are as follows:

1. Create an inverse index that maps normalized forms to UniProt Accession Numbers.

2. Normalize newly extracted terms.

3. Look up the inverse index to find UniProt Accession Numbers of the new terms.

In order to normalize the terms, we employed methods described in [57,64], in which the normalization rules were automatically generated from BioThesaurus [9] (where terms are clustered according to UniProt Accession Numbers). Normalization rules are evaluated according to *ambiguity *and *variability *metrics. *Ambiguity *quantifies how ambiguous terms in the dictionary are, on average, based on the number of terms that share variants with identical spellings. In contrast, *variability *quantifies the average number of variant forms of each term in the dictionary.

As highly ambiguous or variable terms can lead to impaired performance in mapping tasks, normalization rules should aim to reduce ambiguity and variability values as much as possible. In general, a given normalization rule reduces variability, but at the cost of increasing the ambiguity value. Therefore, an ideal normalization rule would maximize the reduction of the variability, whilst simultaneously increasing ambiguity as little as possible. Some examples of simple normalisation rules are as follows:

• Convert all upper case letters to lower case.

• Remove hyphens that occur within terms.

According to the experimental results reported in [64], normalization performance using the above method achieves comparable results to normalization rules that are hand-crafted by domain experts. Dictionary look-up experiments were conducted using a dictionary of human gene/protein names extracted from BioThesaurus [9], together with a list of gene/protein name snippets from the BioCreative II gene normalization task [65], extracted from MEDLINE abstracts. The automatic normalisation method achieved a precision of 0.767 and a recall of 0.633. This compares favourably with the manually constructed heuristic rules reported in [66], which achieved precision and recall values of 0.730 and 0.657, respectively.

For the purposes of the BioLexicon construction, we generated 1,000 normalization rules, using the gene/protein names gathered from existing databases as the dictionary for normalization rule generation (given that these names represented an updated set compared to those of the BioThesaurus). Terms that could not be mapped to any accession number were omitted. Further control of the quality of new term variants was assured by filtering out highly ambiguous terms - those that were mapped to more than 10 accession numbers were discarded.

### Gathering verbs used in biomedical texts

The verbal entries in the lexicon consist both of those extracted from the MedPost dictionary [19], as well as a set of verbs that were manually selected as either highly relevant or specific to the biomedical domain, based on an examination of the literature.

Three types of orthographic variants have also been manually curated for verbs, as follows:

1. British/American spelling variants, e.g. 'acetylise'/'acetylize' or 'harbour'/'harbor'

2. Hyphenation variants, e.g. 'co-activate' and 'coactivate'

3. A combination of the above, e.g. 'co-localise', 'colocalise' (British); 'co-localize', 'colocalize' (American)

A further manual task was to create related entries for each of these verbs. For example, from the verb *absorb*, the entries *absorption *(noun), *absorbent *and *absorbable *(adjective) and *absorbently *(adverb) were added to the lexicon.

For the domain-specific verbs, grammatical and semantic frame information was acquired using a combination of manual and automatic techniques applied to a collection of domain-specific texts, thus ensuring that the behaviour of the verbs stored in the lexicon reflects observed usage within the literature. It should be noted that nominalised verbs such as *absorption *or *transcription*, which appear particularly frequently in biomedical literature, are also of relevance here. These special types of noun are so-called because they have verb-like meanings, and also behave in a similar way to verbs, in that they take arguments. By linking nominalised verbs to their related verbs in the BioLexicon (i.e., *absorb *and *transcribe *in the case of the above examples), the grammatical and semantic information present for verbs can also help to determine and interpret the arguments of the nominalised verbs.

#### Acquiring grammatical frames for verbs

One of the defining features of the process of extracting grammatical frames for inclusion in the BioLexicon is the fact that, according to domain-specific requirements introduced above and in contrast to many general language resources, these frames should account not only for whatever subjects and objects might occur, but also adverbial and prepositional phrases that ultimately indicate semantic information such as locations and timings of biomedical events. Such types of information, as described in the *Background *section, are crucial for proper interpretation and understanding of biomedical events. This explains why the number of extracted grammatical frames for the BioLexicon is much higher than in the SPECIALIST lexicon, which uses a rather limited set of grammatical patterns derived from general language usage.

An automatic process was used to acquire the grammatical frames for the BioLexicon, based on the results of applying the Enju deep syntactic parser [67]http://www-tsujii.is.s.u-tokyo.ac.jp/enju/ to a text collection of over 6 million words (consisting of both MEDLINE abstracts on the subject of *E. coli*, as well as full papers) in order to obtain a structural analysis of each sentence. In particular, we used the version of Enju adapted to biomedical texts [68], which has been shown to perform with an accuracy of 86.87 F-Score on domain-specific text (the GENIA Treebank corpus [69]).

Following the application of the Enju parser, sets of possible grammatical patterns were extracted for each biologically relevant verb. Each pattern consisted of a number of different types of information, as follows:

• The grammatical type of each argument (e.g., ARG1 corresponds to the grammatical subject of the verb, while ARG2 corresponds to the object).

• The type of "filler" for each argument (e.g., noun phrase)

• The semantic type of the argument, (e.g., PROTEIN, DNA), if available. This was obtained by applying the GENIA tagger [70] to the same texts as the Enju parser, and aligning their outputs.

A filtering step was subsequently applied, in which the conditional probability of each grammatical pattern, given the verb, was calculated. Only those patterns whose probability fell above a certain threshold (> = 0.03) were included as grammatical frames in the BioLexicon. Given the large number of potential frames generated, this step was required to filter out "noisy" frames, i.e., those frames containing grammatical arguments that were not typical for the verb, as well as those frames resulting from possible errors of either parsing or pattern extraction. The exact threshold used was determined based on careful examination of the results obtained using different thresholds.

Below is a simplified example frame, showing only grammatical types of each argument (arguments are separated with "#"). This frame accounts for the behaviour of the verb *control *in the sentence *The LysR-type transcriptional regulator CysB controls the repression of hslJ transcription in Escherichia coli*.

ARG1#ARG2#PP-in

In addition to the arguments that represent the subject and the object of the verb, there is a third argument that begins with the preposition *in*. Within the BioLexicon, grammatical frames are normalised for the ordering of arguments within a sentence, as these can vary. For example, the subject and object order varies according to whether the passive or active voice is used, and prepositional phrases can occur in different positions. To allow verbs particularly associated with passive voice usage to be identified, the BioLexicon records the percentage of times that the verb occurs in the passive voice within the corpus. The frame shown above would account for all of the following sentences:

*1. The LysR-type transcriptional regulator CysB controls the repression of hslJ transcription in Escherichia coli*.

*2. In Escherichia coli, the LysR-type transcriptional regulator CysB controls the repression of hslJ transcription*.

*3. The repression of hslJ transcription is controlled by the LysR-type transcriptional regulator CysB in Escherichia coli*.

The inclusion within grammatical frames of specific prepositions that occur at the beginning of particular arguments can be important in linking grammatical and semantic frames. Sometimes, a verb may occur with multiple arguments, each beginning with a preposition. Different types of prepositions typically distinguish different semantic roles, as illustrated in the following sentence: *A promoter has been identified that directs relA gene transcription **towards **the pyrG gene **in **a counterclockwise direction **on **the E. Coli chromosome*. Here, the verb *directs *occurs with 3 arguments beginning with different prepositions, i.e., *towards, in *and *on*, each of which corresponds to a different semantic role in the event, namely DESTINATION, MANNER and LOCATION (see next section for more details on these semantic roles).

The BioLexicon thus includes a range of detailed information about the domain-specific grammatical behaviour of verbs, which, if accessed by parsers, can help to drive the process of determining the most appropriate grammatical analysis. Features such as typical fillers for arguments, in terms of both grammar and semantics (i.e. NE types), can allow sophisticated constraints to be applied during the parsing process. The inclusion of important modifier phrases as part of the grammatical frames can help parsers to ensure that all phrases relevant to the correct interpretation of an event are correctly identified. These may include, for example, phrases that describe the location or environmental conditions of the event. Determining how such phrases are to be interpreted is the job of the semantic frames, which are described in the next section.

#### Acquiring semantic event frames for verbs

Although grammatical parsers such as Enju have reached an appropriately mature level, the same cannot be said for semantic parsers. This meant that a fully automatic process, such as the one described for grammatical frame acquisition, could not be applied to the acquisition of semantic frames for the BioLexicon. Instead, semantic information about verbs was extracted from a corpus of 677 MEDLINE abstracts on the subject of gene regulation, which had been manually annotated (i.e., in a markup language) with information about semantic events relating to gene regulation by a group of domain experts [71]. A further corpus of 240 abstracts (GREC) was annotated with instances of events, for the purposes of evaluating event extraction systems making use of the BioLexicon [72].

Annotation was carried out for both verbs and nominalised verbs (e.g., *regulation*) that describe events relating to gene regulation. For each such verb or nominalised verb, the semantic arguments of the event occurring within the same sentence were labelled with both semantic roles and NE types. A proportion of the abstracts was annotated by more than one annotator, in order to evaluate the consistency of the annotations produced. For the assignment of semantic roles, inter-annotator agreement (i.e. consistency between annotators) of up to 0.89 was achieved (maximum = 1.00). As an aside, a 'perfect score' remains elusive - illustrating neatly that consensus of expert judgement is hard to achieve and that some degree of uncertainty is only to be expected within science [73].

The type of annotation undertaken is exemplified below for the sentence *Fis activates rrnB P1*. The event is centred on the verb *activates *and 2 semantic arguments have been annotated, i.e. *Fis *as the AGENT and *rrnB P1 *as the THEME.

$${[}_{\text{Agent}\_\text{Protein}}\text{Fis}]\;{\text{[}}_{\text{Event}}\text{activates]}\;{[}_{\text{Theme\_DNA}}\text{rrnB P1}]$$

The assignment of NE types (e.g. *Protein*, *DNA*) to biomedical entities or processes from a domain-specific taxonomy can further help in mapping between grammar and semantics, in that restrictions can be placed on the categories of NEs that can constitute a particular type of semantic argument. The NE taxonomy used for annotation is summarised in Table 2.

**Table 2:** Top level NE categories

NE class	Definition
**DNA**	Entities chiefly composed of nucleic acids and their structural or positional references. This includes the physical structure of all DNA-based entities and the functional roles associated with regions thereof.
**PROTEIN**	Entities chiefly composed of amino acids and their positional references. This includes the physical structure and functional roles associated with each type.
**EXPERIMENTAL**	Both physical and methodological entities, either used, consumed or required for a reaction to take place.
**ORGANISMS**	Entities representing individuals or collections of living things and their component parts.
**PROCESSES**	A set of event classes used to label biological processes described in text.

The NE taxonomy used during event annotation is organised into 5 top-level classes, each of which contains a hierarchically structured set of more specific terms. Annotators were instructed to assign the most specific term possible. The general definition of each top-level class is shown in the table.

A set of 13 event-independent semantic roles [71] was used, including both domain-independent and domain-dependent roles. This set of roles (documented in Table 3), which has been tailored to the biomedical domain in consultation with subject experts, is intended to provide a general characterisation of the meaning of each argument, independent of the verb being used. Some of the roles are based on those used within general language resources (e.g., [38]) and are widely traceable across all domains; others are either domain-specific (namely CONDITION and MANNER) or particularly important for the precise definition of complex biological relations, even though not necessarily specific to the field (e.g., LOCATION and TEMPORAL).

**Table 3:** Semantic roles

Role Name	Description	Example ([...] = semantic argument, small capitals = focussed verb)
AGENT	Drives/instigates event	[The narL gene product] ACTIVATES the nitrate reductase operon

THEME	a) Affected by/results from event b) Focus of events describing states	[recA protein] was INDUCED by UV radiation [The FNR protein] RESEMBLES CRP

MANNER	Method/way in which event is carried out	cpxA gene INCREASES the levels of csgA transcription by [dephosphorylation] of CpxR

INSTRUMENT	Used to carry out event	EnvZ FUNCTIONS through [OmpR] to control NP porin gene expression in E. Coli.

LOCATION	Where *complete *event takes place	Phosphorylation of OmpR MODULATES expression of the ompF and ompC genes in [Escherichia coli]

SOURCE	Start point of event	A transducing lambda phage was ISOLATED from [a strain] harboring a glpD''lacZ fusion

DESTINATION	End point of event	Transcription is activated by BINDING of the cyclic AMP (cAMP)-cAMP receptor protein (CRP) complex to [a CRP binding site]

TEMPORAL	Situates event in time/w.r.t. another event	The Alp protease activity is DETECTED in cells [after introduction] of plasmids

CONDITION	Environmental conditions/changes in conditions	Strains carrying a mutation in the crp structural gene fail to REPRESS ODC and ADC activities in response to [increased cAMP]

RATE	Change of level or rate	marR mutations ELEVATED inaA expression by [10- to 20-fold] over that of the wild-type.

DESCRIPTIVE-AGENT	Descriptive information about AGENT of event	HyfR ACTS as [a formate-dependent regulator]

DESCRIPTIVE-THEME	Descriptive information about THEME of event	The FNR protein RESEMBLES [CRP].

PURPOSE	Purpose/reason for the event occurring	The fusion strains were USED [to study] the regulation of the cysB gene

For each semantic role, a brief description is given, together with an example sentence containing an instance of the role. In the example sentences, the verb on which the event is centred is indicated using small capitals, whilst the event argument corresponding to the appropriate semantic role is indicated within square brackets.

Based on the semantic event annotations added to the MEDLINE abstracts, a set of verb-specific semantic frames was extracted for inclusion in the BioLexicon. An example event frame, corresponding to the semantic behaviour of the example sentence above involving the verb *activate*, is as follows:

activate(Agent=>Protein,Theme=>DNA)

For each semantic role, an NE type is used to characterise its possible instantiations, if available.

#### Linking grammatical and semantic frames

In the majority of cases, arguments in semantic frames will have corresponding counterparts in grammatical frames. Given the high level of accuracy of grammatical parsing, the most straightforward way to determine the semantic interpretation of an event is to firstly determine its grammatical arguments, and then to map these to their semantic counterparts. As has been explained in the *Background *section, amongst different verbs, there is no consistent semantic interpretation of a given grammatical argument type. For this reason, the BioLexicon must indicate the exact correspondences between grammatical and semantic arguments for each different verb. Such correspondences were identified via a manual linking process [36]. The linking process was defined by simultaneously taking into account both general and verb-specific information, as follows:

a) general linguistic constraints regarding the alignment of hierarchies of semantic roles and grammatical functions. Given a semantic role hierarchy (agent > theme ...) and a grammatical functions hierarchy (subject > object ...), the mapping usually proceeds from left to right;

b) a list of 'prototypic' grammatical realisations of semantic arguments, as specified in the annotation guidelines followed by annotators during the manual annotation of events (provided in [74]). For example, the AGENT semantic role is typically realised as the grammatical subject of a verb, whilst the INSTRUMENT role typically occurs in a prepositional phrase headed by one of the following prepositions: *with, through, using, via *or *by*.

c) general language repositories of individual semantic frames containing both syntactic and semantic information.

Different types of mapping were performed, depending on whether or not the grammatical and semantic frames contained the same number of arguments. Possible cases were as follows (where ">" separates the semantic role and its corresponding grammatical argument, and "#" separates different arguments).

• Each semantic argument is mapped to a single grammatical argument, e.g., *AGENT > ARG1#THEME > ARG2*. Many sentences with verbs that describe biological processes, having both a subject and an object, would fit this mapping, e.g., *The narL gene product activates the nitrate reductase operon*.

• The grammatical frame contains more slots than the corresponding semantic frame, e.g., *0 > ARG1#THEME > ARG2#DESTINATION > PP-into*. In this mapping, there is no semantic argument that corresponds to the grammatical subject in the grammatical frame. This is typically the case of event frames which do not contain explicit mention of an AGENT role. Such cases apply most frequently to passive sentences such as *The wild-type pcnB gene was cloned into a low-copy number*.

• The semantic frame contains more slots than the corresponding grammatical frame, e.g. *AGENT > ARG1#THEME > ARG2#LOCATION > PP-in#CONDITION > 0*. In this mapping, there is no grammatical argument to which the semantic CONDITION argument can be mapped. There are two possible explanations for this. Firstly, semantic annotation was carried out by biologists without specialist linguistic knowledge. Therefore, semantic annotation was not guided or constrained by the syntactic structure of he sentence in which the event was contained. This means that annotators sometimes identified semantic arguments that were not grammatically related to the verb. Secondly, grammatical parser errors may mean that the grammatical argument corresponding to the CONDITION was not recognised by the parser.

### BioLexicon representation model

Since one of the main aims of the BioLexicon is to foster semantic interoperability of systems in the biomedical community, the ISO (International Organization for Standardization) standard Lexical Markup Framework (LMF) was chosen as the reference meta-model for the structure of the BioLexicon [75]. The LMF, together with linguistic constants used for lexical description (called data categories), provides a common, flexible representation of lexical objects that allows for the encoding of detailed linguistic information. This is particularly important in representing the kinds of grammatical and semantic information that have been acquired for biologically relevant verbs [76], as described above. Finally, the model allows term entries in the BioLexicon to be linked to classes in the GRO [23], which was developed in parallel with the BioLexicon, in order to further facilitate knowledge extraction within the domain.

The BioLexicon is modelled on an XML Document Type Definition (DTD) based on the LMF standard: it implements the core ISO model plus objects taken from the Natural Language Processing (NLP) extensions for morphology, syntax (grammar) and (lexical) semantics. The BioLexicon model, therefore, consists of a subset of the lexical modules and lexical classes of the LMF standard. In particular, it consists of a number of independent lexical objects (or classes) and a set of Data Categories (DCs), i.e., attribute-value pairs, which represent the main building blocks of lexical representation, especially tuned to the design goals of the lexicon.

DCs are a fundamental component of lexical description, which ensure the standardisation of the resource. They represent pieces of linguistic content that are not part of the model proper, but are used to describe the various instances of its objects. The set of DCs used in the BioLexicon consists of both categories drawn from the standard sets of the ISO Data Category Registry [77,78], and categories created specifically for the biomedical domain. In doing this, we also aim at establishing a standard set of Data Categories for this domain. Examples of ISO data categories are *partOfSpeech *and *writtenForm*, whilst *SourceDC *and *confidenceScore *are examples of DCs specifically created for use by the biomedical domain on the basis of expert requirements. *SourceDC*, for instance, is used to encode the original source (be it a database or a corpus) from which a term has been extracted; *confidenceScore*, meanwhile, is a special feature used to encode the confidence value assigned to term variants and subcategorisation information by the learning methods applied to acquire such information from texts.

#### Automatic population of the BioLexicon database

The conceptual model described above has been implemented as a relational database. A key property and an innovation of the BioLexicon Database (BLDB) is that it comes equipped with automatic loading procedures for its population. The BLDB consists of two modules: a MySQL database [79], and a Java [80] software component for the automatic population of the database. The BioLexicon data is encoded in an XML interchange format (XIF), which is fundamental to the automatic population of the BLDB. The XIF data is read by the Java procedures in order to parse, read and load data into the BLDB. The XIF is a simplified version of the BioLexicon DTD, which accommodates the needs of data providers and facilitates the automatic uploading of the database. It therefore facilitates standardization of the data extracted from the different terminological resources and from texts (via text mining techniques) and allows both the uploading procedures and the BLDB to remain independent from native data formats. The database is structured into three logically distinct layers:

1. The 'dictionary layer' contains tables used in the initial handling of the XIF and rules that automatically build SQL instructions to populate target tables;

2. The 'staging layer' is a set of hybrid tables for volatile data; i.e. tables that exist only for a specific purpose in the BioLexicon creation process, but which can be truncated (cleared) after data they contain have been read.

3. The 'target layer' contains the actual BioLexicon tables, i.e. tables that directly instantiate the BioLexicon DTD and contain the final data.

The separation between target tables (the BioLexicon proper) and 'operational' tables allows for the optimization of data uploading into the BLDB and ensures easy extendibility both of the database and of the uploading procedures.

## Results

In this section, we firstly provide some statistics regarding the coverage of the BioLexicon, and compare this coverage to other resources. Subsequently, we show and explain some sample entries from the BioLexicon, in the context of the web interface.

### BioLexicon coverage

The distribution of the ~2.2 M entries in BioLexicon amongst the 4 part of speech categories is as follows:

• Noun - 2,231,574 entries

• Adjective - 3,428 entries

• Verb - 1,154 entries

• Adverb - 550 entries

In the sections below, further statistical details are provided about the entries within the *Noun *and *Verb *categories.

#### Noun coverage

The vast majority of the entries in the BioLexicon belong to the *Noun *category, with most of these entries having been either extracted from existing databases/ontologies or from MEDLINE abstracts using text mining techniques. Table 4 classifies these entries according to the type of entity that they represent.

**Table 4:** Numbers of different types of terms in the BioLexicon

**Semantic Type**	**No. of Entries**	**No. of Variants**	**Semantic Type**	**No. of Entries**	**No. of Variants**
	
Gene/Protein	1,640,608	1,408,312	Diseases	19,457	11,314
	
Chemicals	19,637	77,475	Molecular Roles	8,850	29,831
	
Organisms	482,992	182,610	Cell	842	512
	
Enzymes	4,016	4,164	Transcription Factors	160	129
	
Protein Domains	16,940	15,412	Operons	2,672	368
	
Protein Complex	2,104	418	Sequences	1,431	741

For each type entity, the total number of entries is shown, together with the total number of variant terms identified.

Whilst the majority of the term variants were also extracted from databases and ontologies, 70,105 new variants of gene/protein names were extracted from texts (i.e. abstracts extracted from approximately 15 million MEDLINE records) using the text mining techniques described in the *Methods *section. The large numbers of variants that appear only in texts but not in existing databases provide evidence of the frequency with which new term variants are attested in articles, and thus that automatic text mining methods such as those described are an essential step for improving the coverage of the BioLexicon, and hence enhancing the performance of text mining systems that make use of it.

#### Verb coverage

Several statistics regarding the verbal entries in the BioLexicon are shown in Table 5. Most of the work for verbs has concentrated on generating information relating to the 658 domain-specific verbs that were manually identified. Due to the differing sizes of the text collections used to obtain the grammatical and semantic information about domain-specific verbs, semantic information is not currently available for every such verb recorded in the lexicon. Whilst grammatical information has been generated for all 658 domain-specific verbs, there are currently 168 of these verbs for which semantic information is additionally available. It is from this subset of verbs that the 668 links between the grammatical and semantic frames were manually created.

**Table 5:** Verb statistics

	Domain-specific verbs	General language verbs (from MedPost dictionary)
Total number	658	496

Inflected forms	15,274 (all verbs)	

Related entries (nouns, adjectives, adverbs)	2,764	-

Grammatical frames	1,760	

Semantic event frames	856	-

Grammatical-semantic mappings	668	-

Total verbs with syntactic and semantic frames	168	-

Most information is concerned with the 658 domain-specific verbs, including the curation of related entries, and the production of grammatical and semantic information. Each verb may have several grammatical or semantic frames associated with it, due to the different patterns of arguments that can occur with each verb. This explains why the number of grammatical frames, semantic event frames and grammatical-semantic links is higher than the total number of domain-specific verbs.

#### Comparison of coverage to other lexical resources

**Figure 1:**
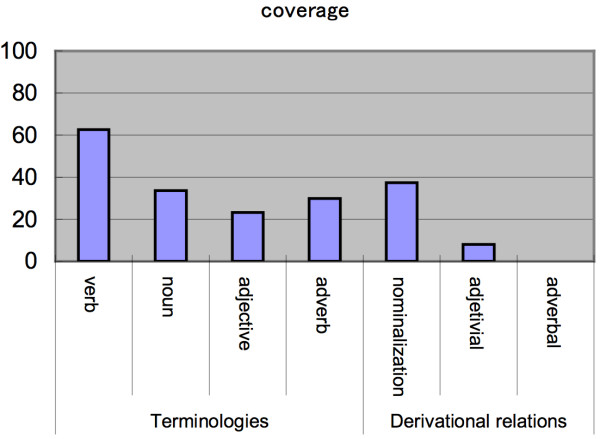
**Word and relation coverage (%) of BioLexicon entries, compared to WordNet**. The percentages shown correspond to the proportion of entries or relations occurring in the BioLexicon that also occur in WordNet.

**Figure 2:**
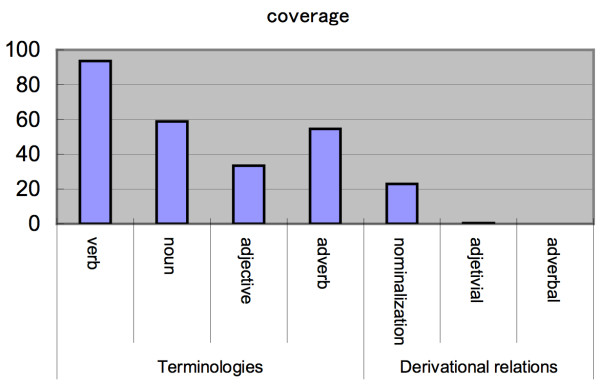
**Word and relation coverage (%) of BioLexicon entries, compared to the SPECIALIST Lexicon**. The percentages shown correspond to the proportion of entries or relations occurring in the BioLexicon that also occur in the SPECIALIST Lexicon.

The coverage of the BioLexicon has been evaluated with respect to existing comparable resources, i.e., WordNet [59] and the SPECIALIST lexicon [18]. WordNet is a large lexical database of English, which contains some domain-specific terms. The SPECIALIST lexicon, which was introduced above, is targeted at the biomedical domain, and includes both words related to this domain, and well as general English words. Figures 1 and 2 compare the coverage of these 2 resources with the BioLexicon, in terms of both the number of terms covered (categorized according to parts-of-speech), as well as the derivational relations from verbs that are covered. These relations consist of nominalizations (e.g., *transcribe *(verb) *-> transcription *(noun)), adjectival derivations (e.g., *transcribe *(verb) *-> transcriptional *(adjective)) and adverbial derivations (e.g., *transcribe *(verb) *-> transcriptionally *(adverb)).

The figures show that, generally, there are a large number of words in the BioLexicon that are not covered by the other resources. For WordNet, this is to be expected, since it is not specifically targeted at the biomedical domain. The differences in the coverage of the SPECIALIST lexicon and the BioLexicon can be partly explained by the fact that the BioLexicon contains vocabulary from areas that are not specifically targeted by the SPECIALIST lexicon, e.g., molecular biology. It is only in the category of verbs that the SPECIALIST lexicon covers most of the entries present in the BioLexicon. However, as explained in the *Methods *section, the BioLexicon provides more detailed and domain-specific information about the syntactic properties of verbs than is provided in the SPECIALIST lexicon. The BioLexicon also deals with semantic aspects of verb behaviour, which are absent from the SPECIALIST lexicon.

Many of the derivational forms covered by the BioLexicon are also missing from the other resources. In this respect, WordNet has a higher coverage than the SPECIALIST lexicon, due to its emphasis on providing systematic links between words. So, for example, the derivation *retroregulate -> retroregulation *is present in WordNet, as well as the BioLexicon. However, since the coverage of domain-specific words in WordNet is not extensive, it is not possible for the coverage of biology-specific derivations to be very high. In the SPECIALIST lexicon, it appears that derivations are not dealt with in a similarly systematic way. Although some nominalizations of verbs are present, the opposite is not always true. For example, the nominalization *retro-regulator *is present in the SPECIALIST lexicon, but the verb *retroregulate *is not included.

We can thus conclude that the BioLexicon constitutes a text mining resource that complements both WordNet and the SPECIALIST lexicon, in that it contains a large number of vocabulary items that are not covered by the other 2 resources, in addition to a large number of derivationally-related words.

#### Evaluation of coverage against gold standard data

As a further means to determine the coverage of the terminology in the BioLexicon, we have evaluated the extent to which biomedical named entities annotated in a gold standard corpus are found in the BioLexicon. In order to perform the evaluation, we compared the performance of two dictionary-based POS taggers in recognising protein names in the JNLPBA-2004 training dataset [61]. Using such taggers, words and word sequences that correspond to terms in the dictionaries are treated as a single, complete unit, and flagged as corresponding to biomedical terms, through the assignment of a tag such as *NN-BIOMED*. Since the POS tag *NN *corresponds to a noun, *NN-BIOMED *is used denote a biomedical noun or term. The advantage of using dictionary-based taggers can be appreciated when considering a protein name such as *met protooncogene precursor*. As *met *is also the past tense of the verb *meet*, a POS tagger without domain-specific knowledge is likely to make tagging errors.

The two dictionary-based taggers used are the BLTagger [81], which makes use of information in the BioLexicon, and a tagger which was built during the development of our NER tool [58], and whose dictionary makes use of protein names extracted from the BioThesaurus (we will refer to this as the BT-Tagger). The performance of the two taggers in recognizing protein names in the JNLPBA-2004 test dataset is shown in Table 6.

**Table 6:** Evaluation of BioLexicon Coverage on JNLPBA-2004 dataset

	BLTagger	BT-Tagger
Full	55.54	47.96

Left	56.72	55.72

Right	59.24	55.63

The table compares the performance of two dictionary-based POS taggers, the BLTagger and the BT-Tagger, in recognising protein names in the JNLPBA-2004 test dataset. For each tagger, F-scores are reported in terms of full matches (i.e., the sequence of words identified as a biomedical noun by the tagger matches exactly the text span annotated as a protein in the JNLPBA-2004 training dataset) as well as right and left boundary matches (i.e., the sequence of words identified as a biomedical noun by the tagger matches either the left or right boundary of the corresponding annotation in the JNLPBA-2004 training data).

The table shows that the BLTagger performs considerably better than the BT-Tagger, with more than a 7.5% increase in the correct recognition of full protein names. With the BLTagger, the discrepancies between the cases where the tagger correctly recognized the full protein name and those where only one of the boundaries (left or right) was correct are smaller than the discrepancies achieved by the BT-Tagger.

As will be recalled, the inventory of proteins contained within the BioLexicon is largely based on those in BioThesaurus, but with two main differences:

1. Steps were taken to remove potential noise from the BioThesaurus

2. Protein name variants were extracted from the literature using text-mining techniques.

Our results clearly demonstrate that the BioLexicon has improved coverage of protein names, compared to BioThesaurus. The results also suggest that entries in the BioLexicon are more likely than those in BioThesaurus to correspond to complete protein names encountered in biomedical documents.

It should be understood that looking up names of biomedical terms in the BioLexicon should not be seen as an alternative to the training of NER systems. As reported in [58], the results of simple dictionary-based recognition of protein names (using the BT-Tagger) can be increased by over 25% if an NER system is trained to take into account not only dictionary-based information, but also other features in the surrounding text. Therefore, incorporating a better coverage lexical resource such as the BioLexicon into NER systems has the potential to further increase their performance.

### Sample entries

This section aims to provide more specific examples of the types of information that are available in the BioLexicon. For ease of understanding, this explanation is facilitated through the provision of a number of screenshots from the BioLexicon web access interface. It should, however, be noted that the interface is provided for human interpretation of the information within the lexicon. As such, these illustrative screenshots cannot do full justice to the underlying complex and sophisticated of the information available in the BioLexicon web interface. Normally, an application would access the BioLexicon database programmatically via an API or web services.

#### Sample verb entry

**Figure 3:**
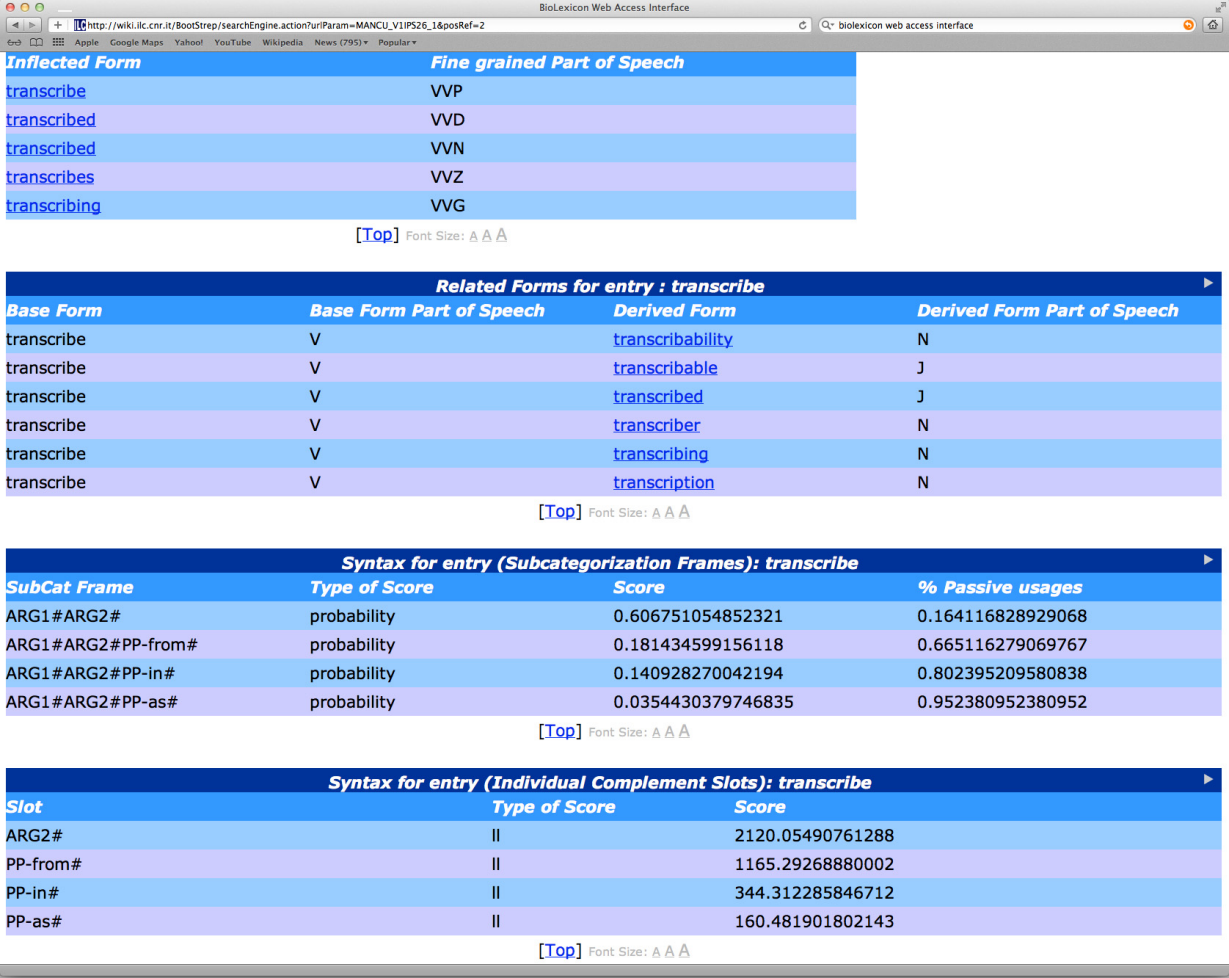
**Grammatical information provided for the verb *transcribe***. The first table displays the different possible inflections of the verb (e.g., different tenses), whilst the second table shows words belonging to different parts of speech that are derived from the verb (e.g., the noun *transcriber *and the adjective *transcribable*). The third and fourth tables provide syntactic (grammatical) information about the behaviour of the verb.

**Figure 4:**
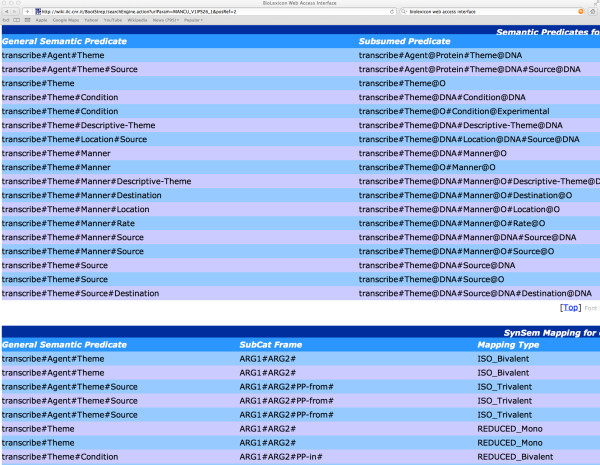
**Semantic information provided for the verb *transcribe***. The top table provides information about the different patterns of semantic roles that can occur with the verb, whilst the bottom table shows correspondences between grammatical and semantic frames.

**Figure 5:**
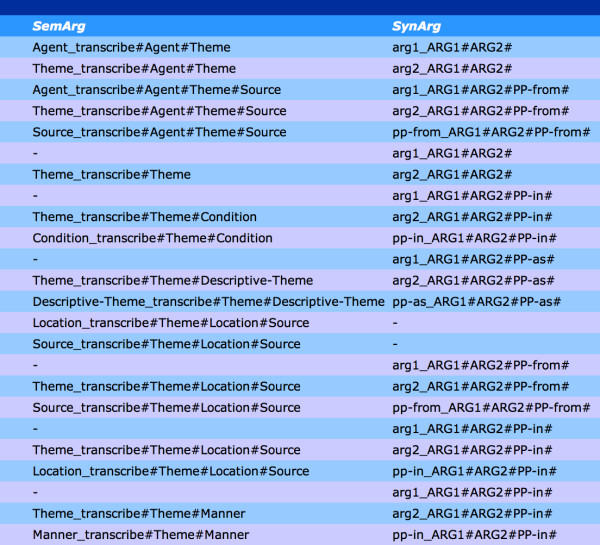
**Grammatical-semantic linking information between individual arguments for the verb *transcribe***. Each row represents a link between a semantic argument (shown before the underscore in the *SemArg *column) and a grammatical argument (shown before the underscore in the *SynArg *column), in the context of a particular pair of linked semantic and grammatical frames, which are shown after the underscores.

Figures 3, 4, and 5 show screenshots of different types of information that is stored in the BioLexicon for the verb *transcribe*. Figure 3 displays some of the grammatical information available for the verb, whilst Figure 4 displays some of the information that is provided regarding the semantic side of the verb's behaviour. Figure 5 displays information regarding the linking between grammatical and semantic behaviours.

The third table in Figure 3 displays the possible complete grammatical patterns that can occur with *transcribe*. All of these contain ARG1 and ARG2 (corresponding to the grammatical subject and object of the verb, respectively), possibly accompanied by a phrase beginning with a preposition (either *from, in *or *as*). Examples are as follows:

• *Short RNAs are transcribed **from **repressed polycomb target genes*.

• *When transcribed **in **the presence of RNase A..*.

• *The ribosomal spacer in Xenopus laevis is transcribed **as **part of the primary ribosomal RNA*.

For each grammatical pattern, two statistics are provided: firstly, the probability of each pattern, given the verb. The most likely pattern for the verb is for only ARG1 and ARG2 to be present, with a probability of 0.60. This pattern is over 3 times more likely to appear than the pattern in which a PP-*from *is present (i.e., a prepositional phrase beginning with *from*). The second statistic provides the probability that the verb will occur in the passive voice, as in all of the examples provided above. From this table, it can be seen that the pattern involving *PP-as *almost always occurs in the passive voice (95% of the time). However, when only ARG1 and ARG2 are present, it is more common for the active voice to be used, as in *RNA pol II transcribes mRNA and the small nuclear RNA (snRNA)*. Also provided, but not shown in the figure, is information about the form that each type of grammatical argument can take (e.g., noun phrase).

The fourth table in Figure 3 provides another statistical measure, the log likelihood (ll), for each possible grammatical argument that can occur with the verb. This measure attempts to quantify how strongly associated each argument is with the verb. It is intended to complement the probability information provided in the third table, to allow more informed decisions to be made when analyzing the grammatical structure of a sentence, e.g., to determine how likely it is that particular phrases that occur in the same sentence as the verb should actually be attached to the verb.

In Figure 4, the left hand column of the table at the top of the figure displays the different patterns of semantic roles that can occur with the verb. The right-hand column provides possible "fillers" for these roles, in terms of NE categories. For example, in the pattern involving an *Agent *and a *Theme*, it is probable that the *Agent *will be NE of type *Protein*, whilst the *Theme *will be an NE of type *DNA*.

The second table in Figure 4 shows correspondences between semantic and grammatical frames. So, for example, the fourth line in the table shows that the semantic frame *transcribe#Agent#Theme#Source *(i.e., the frame with three semantic arguments with the roles of *Agent, Theme *and *Source*) is grammatically realized in texts though the pattern *Arg1#Arg2#PP-from#*, i.e., a pattern consisting of a subject, an object and a *PP-from *(but not necessarily occurring in that order in the text). As described in the *Methods *section, there is no consistent correspondence between syntactic argument types and semantic roles. Therefore, a different part of the table (shown in Figure 5) provides correspondences between individual semantic and grammatical arguments. If we consider the first line of this table, this shows that the *Agent *semantic argument within the semantic frame *transcribe#Agent#Theme *corresponds grammatically to *Arg1 *in the grammatical frame *Arg1#Arg2#*, i.e., the *Agent *role in an event describing a transcription will normally correspond to the subject of the verb. Similarly, line 5 of the table tells us that a *PP-from *phrase that is grammatically associated with the verb will play the semantic role of *Source*, i.e., the location from which the transcription took place, as exemplified in the following sentence: S*hort RNAs are transcribed **from repressed polycomb target genes***.

#### Sample noun entry

**Figure 6:**
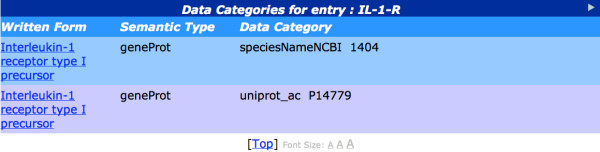
**Source database id information for the term *Interleukin-1 receptor type I precursor***. There is a corresponding entry in both the NCBI species name database and the UniProt database.

**Figure 7:**
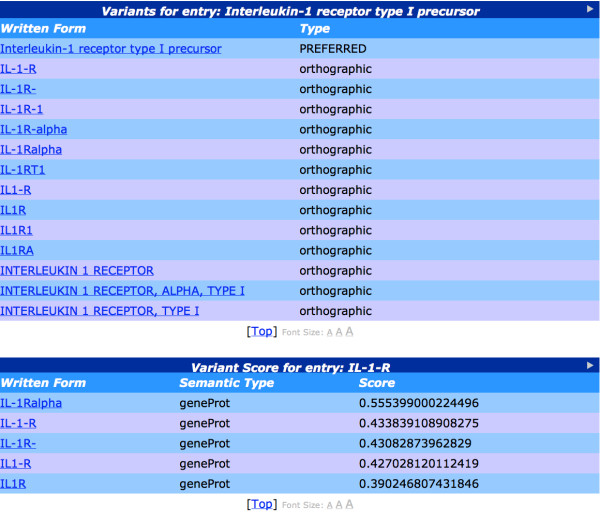
**Variants of the term Interleukin-1 receptor type I precursor**. The top table shows all variants of the term stored in the BioLexicon, together with the type of variant (e.g. orthographic). The entries in the bottom table correspond to those entries that have been automatically extracted from texts, together with confidence scores.

Information from the entry for *Interleukin-1 receptor type I precursor *is shown in Figures 6 and 7. In Figure 6, the ids of the term in different source databases are shown. Figure 7 displays the textual variants of the term that are stored in the BioLexicon, drawn either from one of the source databases, or else through the application of the automatic text mining methods. The bottom table in the figure displays the variants that have been automatically extracted from texts, and automatically mapped as variants according to string similarities, as described in the *Methods *section. The scores provided correspond to the confidence with which the mapping has been made.

## Discussion

Text mining systems that exploit the BioLexicon can gain help with a number of key steps in the processing of biology texts, i.e.:

1. Domain-specific POS tagging.

2. Domain-specific lemmatisation (i.e., finding the base forms of words, such as singular forms of nouns, or infinitive forms of verbs).

3. Mapping from textual variants of terms/named entities to an appropriate accession number from the reference data resource.

4. Identification of key terminological verbs.

5. Identification of which terms/NEs stand in key grammatical relationships to the identified verbs.

6. Identification, via frame linking, of the semantic roles that the terms/NEs in these grammatical relationships are playing.

7. Establishment of the existence of particular types of semantic event in a text, with their participants being grounded, via frame linking, in the actual words of the text, allowing the scientist to then 'see the evidence' for some proposed event instance.

In the *Results *section, we have already introduced the BLTagger, which can be used to carry out domain-specific POS tagging. In this section, we provide further examples of how the BioLexicon can be exploited in some of the steps described above.

### Using the BioLexicon for lemmatisation of biomedical text

The BioLemmatizer [82] has been developed to perform lemmatisation on biomedical text. It consists of two parts - a lexicon that covers inflected word forms and their corresponding lemmas in both the general English and biological domains, and a set of rules that express morphological transformations, in order to handle words that are not present in the lexicon. BioLemmatizer's lexicon augments MorphAdorner's lemmatisation lexicon for general English [83] with information specific to the biomedical domain, extracted from both the BioLexicon and GENIA lexical resources [70].

In [82], the performance of BioLemmatizer on the CRAFT corpus development set [84] is compared with 8 other tools that perform lemmatisation. All 9 tools agreed on the lemmatisation of about 71% of the 6,775 unique token in the corpus. For the remaining 29% of the corpus, BioLemmatizer significantly outperformed the other tools, achieving an F-score of 96.37. The other tools ranged from 50.60 to 81.90 F-score, with MorphAdorner constituting the second-best tool.

A further set of experiments evaluated the component parts of BioLemmatizer on the same 29% of the corpus. Using MorphAdorner lexicon as the only resource for lemmatisation resulted in an F-Score of 53.52, demonstrating that general-language resources alone are not sufficient to perform accurate lemmatisation of biomedical texts. Augmenting the MorphAdorner lexicon with information from the BioLexicon resulted in a significant increase in performance, to 64.43 F-score.

### Using the BioLexicon in information retrieval

Information Retrieval (IR) is the process of retrieving documents that are relevant to a user's query. It is both a component in standard text mining processes and also an activity in its own right for the human seeking to satisfy an information need. In searching for biomedical documents, it is often the case that queries contain long multiword technical terms that should be handled as single expressions. As the BioLexicon contains such complex terms, it should be able to help in such situations.

In order to test this hypothesis, we conducted evaluation experiments on the Text Retrieval and Evaluation Conference (TREC) 2007 Genomics Track (http://ir.ohsu.edu/genomics/) 'gold standard' data [4] and calculated the document mean average precision (MAP) in accordance with TREC specifications. This measure is to be interpreted as follows: any document identifier that has a passage associated with a topic identifier in the set of gold standard passages is considered a relevant document for that topic. (The goal of the TREC 2007 Genomics Track was to generate a ranked list of passages for 36 questions that relate to biological events and processes).

We performed a set of experiments (as reported in [4]) in which query terms were formulated both with and without the use of information from the BioLexicon. All experiments involved initial pre-processing of the free-text queries to remove information-free words (called stopwords) such as *the *or *of*. Following this step, lists of query terms were formulated. As an example, let us consider a query that contains the words *T-cell growth factors*. The following query terms were computed:

• All uni-grams (i.e., single tokens, e.g., *T-cell*), bi-grams (i.e., sequences of 2 words, e.g., *T-cell growth*) and tri-grams (i.e., sequences of 3 words, e.g., *T-cell growth factors*) were extracted from the queries.

• Terms within the query corresponding to entries in the BioLexicon.( e.g., *T-cell growth factors*) were determined.

Experiments involved the formulation of different IR queries. These contained various sets of query terms corresponding to different combinations of all uni-grams, bi-grams and tri-grams in the free-text query, both with and without the addition of terms present within the BioLexicon. Without information from the BioLexicon, the best results were achieved when only word uni-grams were submitted as query terms, which resulted in a document MAP of 0.2744. Adding bi-grams and tri-grams to the list of query terms significantly degraded performance. For example, submitting queries containing both uni-grams and bi-grams found in the query reduced the document MAP score to 0.2257. However, performance of the system was enhanced by adding BioLexicon terms to the list of uni-grams. In this case, the document MAP score increased to 0.2763. The same experiment was conducted using terms found in the SPECIALIST Lexicon instead of the BioLexicon, which resulted in a lower document MAP score of 0.2759. These results demonstrate that the use of technical terms contained within the BioLexicon can effectively improve genomics IR performance.

There were 27 groups that participated in the TREC 2007 Genomics Track. According to assessment using the document MAP measure, the results achieved by the top 6 systems are shown in Table 7. The relative performance of our system (using the configuration with uni-grams and BioLexicon terms) is also indicated in the table. From the table, it can be seen that our system performs with comparable accuracy to the 5th ranked system, and better than the 6th ranked system. Although the differences between the performances of these 3 systems are small, this can equally be said of all systems in the top ranking set. Due to the many differences in approaches and features used, leading to roughly comparable results, it is difficult to determine whether these small differences in performance are statistically significant [85].

**Table 7:** TREC Genomics track 2007 system performance

System	Document MAP
NLMinter (1st)	0.3286

NLMfusion (2nd)	0.3105

MuMshFd (3rd)	0.2906

MuMshFdRsc (4th)	0.2880

UniNE1 (5th)	0.2777

BioLex	0.2763

UniNE3 (6th)	0.2710

The table shows the performances of the top 6 systems (in terms of Document MAP scores) taking part in the TREC Genomics track 2007, together with the performance of our system (configured to use uni-grams and BioLexicon terms as query terms) when applied to the same data. The rankings of the systems taking part in the original challenge are shown in brackets.

It should be noted that the experiments are quite simple, and have the aim only of demonstrating that adding technical terms from the BioLexicon to other query terms can improve genomics IR performance. Whilst the addition of the BioLexicon terms results only in a small improvement over the use of uni-grams alone, the small differences between the performances of other systems mean that any method that can improve performance should be taken seriously. Whilst there were systems that performed slightly better than the BioLexicon-based system, they made use of other external resources such as thesauri and ontologies. Therefore, a method that combines the use of the BioLexicon with other external resources may further enhance performance, and is worthy of further investigation.

### Using the BioLexicon in information extraction

Whilst IR retrieves relevant documents, Information Extraction (IE) [86] processes the documents themselves to locate and structure important pieces of knowledge (i.e., events) contained within them. Within a text mining system, IR can be followed by IE, i.e., once relevant documents have been located, they can be further processed to extract relevant knowledge from them.

We have chosen to exploit the BioLexicon within a challenging IE context, namely that of full parsing, as part of the UKPubMedCentral (UKPMC) text mining services (http://ukpmc.ac.uk/), to locate and extract facts related to the biology domain. Searching for facts within the UKPMC document set is based on indexing three types of information:

1. Deep parsing results (predicate-argument structures) from the Enju parser applied to full papers from the PubMedCentral repository

2. NER results, which are obtained from a tool that is based on the one used to locate candidate term variants for inclusion in the BioLexicon [58], but augmented with additional information from external databases to facilitate the recognition of not only gene and protein names but also other types of entities including diseases, drugs, and metabolites [87].

3. Information about verb relations in the biology domain and their co-occurrence with different patterns of arguments and modifiers. This information is extracted from the BioLexicon.

The BioLexicon is used as the keystone of an IE method applied to the UKPMC collection. In practice, there are three components in the fact extraction process. Firstly, grammatical arguments of verbs in the texts are located through the application of the Enju parser. Only those verbs that are included in the BioLexicon are considered as potential textual "anchors" of events. This seems a sensible first filtering step, given that that BioLexicon is specifically designed to include only domain-specific and domain-relevant verbs, which could potentially describe biomedical events. These candidate events are further narrowed down by selecting only those events in which an NE relevant to the domain is involved in one of the arguments associated with the verb, as it is to be expected that biomedical events will count amongst their participants at least one biologically relevant entity. As a final test, the grammatical argument pattern of the verb should match one of the predicted patterns in the BioLexicon.

Whilst the primary use of the BioLexicon information in this context is as a filter, it also has a boosting effect on the range of facts to be considered. This is because modifier phrases (e.g., those which begin with prepositions) are explored, which would not be considered without its input. Where these modifier phrases contain recognised NEs, this can provide enough evidence for the extraction of a fact that would not otherwise be recorded. Consider the following example:

The pXPC3 plasmid codes for an XPC cDNA that is truncated by 160 bp from the N terminus compared with the wild-type XPC cDNA

Although the Enju parse result treats *code *as an intransitive verb (i.e., without a grammatical object), the information present in the BioLexicon allows the THEME role to be assigned to the prepositional phrase beginning with *for*.

The application of the fact extraction method described above to the UKPMC corpus is ongoing work, given that the corpus itself is expanding year on year, and currently contains 1.8 M research papers. However, we are able to provide a substantive evaluation based on a sample of the corpus. This evaluation is based on a current version of the fact extraction process, applied to publications from three different years, i.e., 2001, 2002 and 2004. The total number of documents processed for these 3 years numbers approximately 80,000, containing 42,778,689 instances of verbs, of which 26,861,273 (62.8%) are lexical (i.e. non-auxiliary) verbs.

Statistics regarding the evaluation are shown in Table 8. Firstly, by eliminating verbs that are not present in the BioLexicon, between 61.5% and 64.8% of the original set of lexical verbs remain (according to year). This illustrates the initial filtering effect of the BioLexicon. Following application of the additional NE-based and grammatical filters, between 17.7% and 27.5% of the total number of verbs that were found in the BioLexicon remain. The strongest of the two filters applied to the remaining verbs is the NE-based filter. According to the year, between 69.9% and 80.2% of verbs appearing in BioLexicon do not any contain NEs within their arguments. These results suggest that many instances of verbs that match BioLexicon entries do not describe biomedical events, and hence the application of such a filter is important to ensure that only verbs that describe relevant facts are extracted. As future work, we plan to investigate whether the NE selectional restrictions specified for particular arguments within BioLexicon semantic frames could complement this filtering process.

**Table 8:** Using the BioLexicon for fact extraction

Year	Total lexical verbs	Verbs in BL (% lex. verbs)	Facts extracted (% BL verbs)	Gramm. frame mismatch (% BL verbs)	Absence of NE in args (% BL verbs)	Facts with prep. args (% of facts)
2001	6,637,052	4,083,325	1,000,571	89,719	2,993,038	187,493
		(61.5%)	(24.5%)	(2.2%)	(73.3%)	(18.7%)

2002	13,412,793	8,694,065	2,417,809	194,986	6,081,289	493,962
		(64.8%)	(27.8%)	(2.2%)	(69.9%)	(20.4%)

2004	6,811,428	4,201,550	742,621	89,249	3,369,690	129,600
		(61.7%)	(17.7%)	(2.1%)	(80.2%)	(17.5%)

The table provides statistics regarding the use of the BioLexicon to identify biomedical facts in approximately 80,000 full text articles taken from the UKPMC corpus. For each of the 3 years represented in this sub-corpus, the total number of lexical (i.e., non-auxiliary) verbs for that year is shown. In the next column, the number of these lexical verbs that match with entries in the BioLexicon (BL) is provided. The figure is also shown as a percentage of the total number of lexical verbs. Next to this is the total number of verbs that are extracted as representing facts (i.e., following the filtering steps), displayed both as an absolute figure and as a percentage of the total number of verbs in the BL. The next two columns display the number of verbs filtered out during the two filtering steps: firstly, the verbs whose grammatical frame does not match with the ones specified in the BL, and secondly, verbs whose arguments do not contain any NEs. The final column displays the number of facts extracted that have one or more arguments corresponding to prepositional phrases, both as a raw figure and as a percentage of the total number of facts extracted.

The filter that removes those verbs whose grammatical frames do not match those specified in the BioLexicon generally only removes a small number of verbs (consistent across the different years at just over 2%). This result demonstrates that domain-specific verbs behave largely as specified in the BioLexicon, thus providing strong evidence that it is a reliable resource to aid in the grammatical processing of biomedical texts.

A further interesting statistic concerns the number of facts extracted that have arguments corresponding to prepositional phrases. Such facts constitute up to 20% of the total facts extracted. This demonstrates the boosting effect achieved by using the domain-specific grammatical patterns in the BioLexicon. Without this information, these arguments, which are often vital for the correct interpretation of the event, would not have been identified.

These preliminary results provide compelling evidence that the grammatical information provided in the BioLexicon can complement other text mining components in building powerful tools for fact extraction within the biomedical domain.

The new UKPMC *EvidenceFinder *search service makes use of the facts that have been extracted from the UKPMC document collection, using the method described above. The service, which is currently available for testing on the UKPMC labs website (http://labs.ukpmc.ac.uk/), allows users to search for information in a more efficient and focussed way than is possible using traditional keyword search.

**Figure 8:**
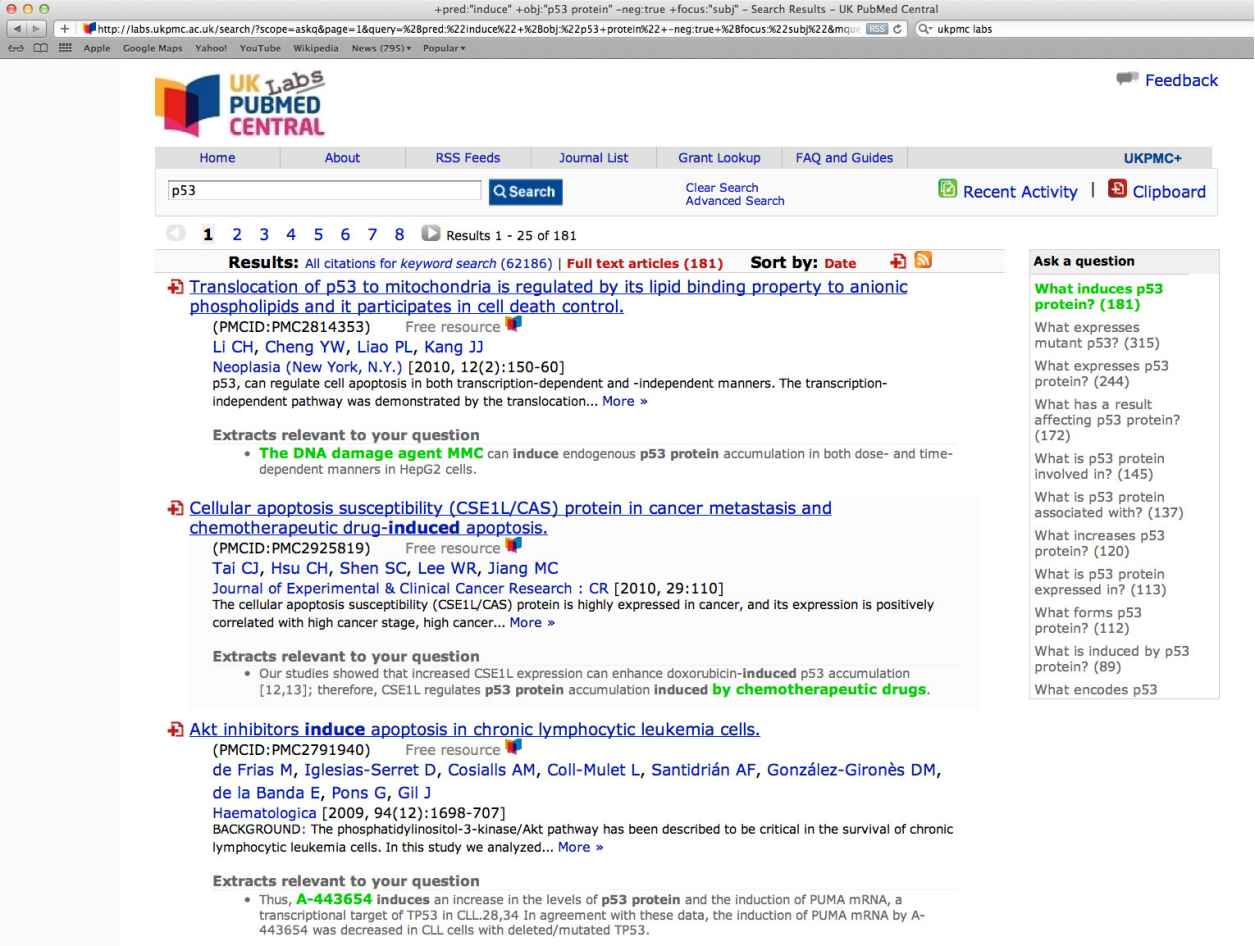
**UKPMC EvidenceFinder interface**. The screenshot displays the results of entering the search term *p53*. Questions corresponding to the facts found in the UKPMC document collection that involve p53 are shown on the right-hand side of the screen. Beside each question is a number, which denotes the total number of documents containing the corresponding type of fact. The currently selected question, i.e., *What induces p53 protein*, is highlighted in green. On the left-hand side of the screen, the summary of each document retrieved by the search concludes with *Extracts relevant to your question*, which lists the sentence(s) within the document that contain facts relevant to the selected question. In each sentence, the answer to the question is highlighted in green, whilst the original search term, and the verb relevant to the question are emboldened. A *Feedback *link at the top of the age makes it easy for users to report problems or suggest new features.

For any given named entity, e.g., *p53*, there can be many different types of fact that mention the entity. Often, a user is only interested in a specific subset of these facts, e.g., those that mention specific types of relationships between p53 and other entities. Given such a search term, EvidenceFinder filters the search results by presenting a list of questions that illustrate the most frequent types of relationship in which the search entity is involved, e.g., *What expresses p53 protein?, What induces p53 protein?, What binds to p53 protein? *These questions are generated from the set of facts extracted from the UKPMC document collection that involve the search term (thus, these are generated questions with known answers, not auto-completed questions that may have no answer). When a question is selected, documents containing corresponding facts are displayed. Sentences containing facts corresponding to the selected question are displayed as part of the search results for each document, with answers related to the question clearly highlighted in each case. An example of the questions and search results generated is illustrated in Figure 8.

As we do not currently have a corpus annotated with gold-standard fact annotations, and as the production of such a corpus would be costly in terms of manual annotation effort required, we are not currently able to report on precision and recall values for the results obtained. However, it should be noted that the engagement and satisfaction of users is of paramount importance in the UKPMC project. Therefore, users have been invited to test and evaluate thoroughly the new EvidenceFinder feature, and feedback will help to assess the quality of the extracted facts.

## Conclusions

This article has presented the BioLexicon, a unique resource comprising rich linguistic information that can support a wide variety of tasks relating to IR and text mining within the biomedical domain, ranging from low-level tasks such as POS tagging to advanced tasks such as extraction of events. As a terminological resource, the BioLexicon brings together terms previously present only in a range of disparate resources, in order to create a more unified, wide-scope resource for use in biomedical text mining. Variants of terms are linked together via accession numbers from reference data resources, e.g., UniProt, ChEBI, NCBI taxonomy and others. Further variants of terms for genes and proteins that appear in the literature, but not in existing databases, have been automatically recognised and mapped to existing terms using text mining techniques. This is important, given the significant number of term variants that appear in the literature, and ensures that the BioLexicon contains many more term variants than can be reasonably included in manually curated resources. We have shown that the BioLexicon covers a large amount of domain-specific vocabulary that is missing from comparable computational lexical resources (i.e., WordNet and the SPECIALIST lexicon), and we have also demonstrated that the coverage of protein names is superior to BioThesaurus.

The wide coverage of terminology in the BioLexicon makes it suitable for a variety of tasks. In terms of IR systems, search interfaces can be improved by providing dynamic completion of query terms that are being typed in, through reference to the lexicon. Retrieved results can also be improved by using the lexicon to find the known variants of search terms entered, thus allowing a greater number of potentially relevant documents to be retrieved.

A further key feature is the semi-automatic acquisition of information regarding the grammatical structure and semantic role of verbs. The corpus-driven nature of the acquisition process ensures that the BioLexicon provides accurate information regarding the observed behaviour of verbs within domain-specific texts. This in turn can help to facilitate the identification of events, together with their participants and the semantic roles assigned to them. Such comprehensive information is not currently available in any comparable domain-specific resource.

In order to foster interoperability, the BioLexicon is modelled using the Lexical Markup Framework, an ISO standard. An XML interchange format (XIF) facilitates integration and standardization of the data extracted from the different terminological resources and from texts via text mining techniques.

We have described how the BioLexicon can be exploited in a number of key tasks relating to the processing of biomedical texts, including POS tagging, lemmatisation, IR and focussed fact extraction from large collections of full papers and abstracts. As future developments, we will integrate the BioLexicon into further text mining applications [81], and will increase its coverage by adding more NEs and terms relevant to the biomedical domain, as well as acquiring grammatical and semantic information for additional verbs from biomedical corpora. We also plan to define a (semi)-automatic process for linking together the syntactic and semantic verb frames. This will allow us to investigate the integration of the BioLexicon within rule-based parsers, which will enable sophisticated constraints to be applied during the parsing process.

Given the rate at which biomedical literature is being published, keeping the BioLexicon as up-to-date as possible is a major concern. The sheer size of the resource means that such a task could not be accomplished without the use of some automatic techniques. Accordingly, as has been described above, partial automatic updating of the BioLexicon can be achieved through periodic execution of the text mining mechanism to identify new term variants appearing in newly published texts.

It is important that the BioLexicon remains a high-quality resource, in order to continue in helping to improve the performance of text mining systems in the biomedical field. Whilst the state-of-the-art in text mining is sufficient to support the automatic update of the resource, experience shows that the best method of maintaining high quality at a reasonable cost is to use a combination of automatic and manual methods. Given the role of human intervention, assistance from the biomedical text mining community will be important in the maintenance of the resource. Already planned initiatives, such as the establishment of a user group for the BioLexicon, will enable the community as a whole to help in ensuring that the lexicon remains a relevant and valuable resource to assist in a wide range of text mining tasks within the domain.

## Authors' contributions

PT, JM, SM and SA were responsible for drafting the manuscript. SA supervised all work undertaken at the University of Manchester, including the semantic event annotation and the discovery/mapping of new terms found in texts. NC supervised all work undertaken at ILC-CNR, including the design of the BioLexicon representation model and population of the lexicon, and the acquisition of linguistic information for verbs. DRS supervised the work undertaken at the EBI, including the extraction of terms from biomedical reference data resources and their semantic relations, and the definition of the XML interchange format (XIF), which was undertaken by PP and VL. YS worked on the curation of terminological verbs and their derivational forms and the automatic extraction of term variants from MEDLINE abstracts and the evaluation of the BioLexicon in the IR context. PT, JM, SM, GV and YS were involved in the design of the semantic event annotation scheme, whilst PT undertook the supervision of this annotation. SM was responsible for all aspects of acquiring linguistic information for verbs. GV carried out the mapping between the grammatical and semantic frames, and SMa implemented the subcategorization extraction software. MM and VQ were responsible for the design of the BioLexicon representation model and RG worked on the implementation of the database model. CR worked on the fact extraction (IE) process in the UKPMC project that makes use of the BioLexicon, undertook the evaluation of the BioLexicon in this context, and worked on the evaluation of the term coverage in the BioLexicon against the gold-standard JNLPBA-2004 dataset. All authors read and approved the final manuscript.
